# The Embodied Brain of SOVEREIGN2: From Space-Variant Conscious Percepts During Visual Search and Navigation to Learning Invariant Object Categories and Cognitive-Emotional Plans for Acquiring Valued Goals

**DOI:** 10.3389/fncom.2019.00036

**Published:** 2019-06-25

**Authors:** Stephen Grossberg

**Affiliations:** Center for Adaptive Systems, Graduate Program in Cognitive and Neural Systems, Departments of Mathematics & Statistics, Psychological & Brain Sciences, and Biomedical Engineering, Boston University, Boston, MA, United States

**Keywords:** invariant object category learning, spatial navigation, visual search, working memory, reinforcement learning, motion perception, attention, adaptive resonance theory

## Abstract

This article develops a model of how reactive and planned behaviors interact in real time. Controllers for both animals and animats need reactive mechanisms for exploration, and learned plans to efficiently reach goal objects once an environment becomes familiar. The SOVEREIGN model embodied these capabilities, and was tested in a 3D virtual reality environment. Neural models have characterized important adaptive and intelligent processes that were not included in SOVEREIGN. A major research program is summarized herein by which to consistently incorporate them into an enhanced model called SOVEREIGN2. Key new perceptual, cognitive, cognitive-emotional, and navigational processes require feedback networks which regulate resonant brain states that support conscious experiences of seeing, feeling, and knowing. Also included are computationally complementary processes of the mammalian neocortical What and Where processing streams, and homologous mechanisms for spatial navigation and arm movement control. These include: Unpredictably moving targets are tracked using coordinated smooth pursuit and saccadic movements. Estimates of target and present position are computed in the Where stream, and can activate approach movements. Motion cues can elicit orienting movements to bring new targets into view. Cumulative movement estimates are derived from visual and vestibular cues. Arbitrary navigational routes are incrementally learned as a labeled graph of angles turned and distances traveled between turns. Noisy and incomplete visual sensor data are transformed into representations of visual form and motion. Invariant recognition categories are learned in the What stream. Sequences of invariant object categories are stored in a cognitive working memory, whereas sequences of movement positions and directions are stored in a spatial working memory. Stored sequences trigger learning of cognitive and spatial/motor sequence categories or plans, also called *list chunks*, which control planned decisions and movements toward valued goal objects. Predictively successful list chunk combinations are selectively enhanced or suppressed via reinforcement learning and incentive motivational learning. Expected vs. unexpected event disconfirmations regulate these enhancement and suppressive processes. Adaptively timed learning enables attention and action to match task constraints. Social cognitive joint attention enables imitation learning of skills by learners who observe teachers from different spatial vantage points.

## 1. Perception, Learning, Invariant Recognition and Planning During Search and Navigation Cycles

**FIGURE 1:**
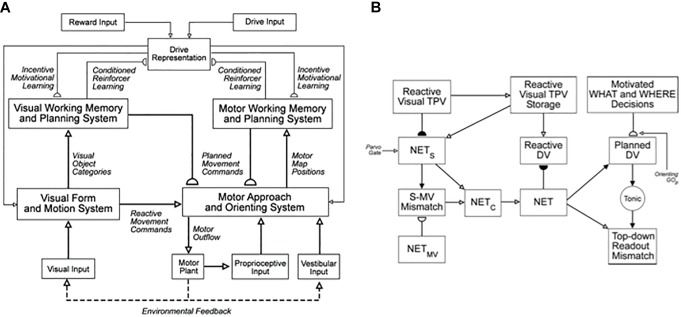
**(A)** The main interactions between functional systems of the SOVEREIGN model. **(B)** The Motor Approach and Orienting System flow diagram depicts the control hierarchy that generates outflow motor commands. See the text for details [Reprinted with permission from Gnadt and Grossberg (2008)].

This article contributes to an emerging scientific and computational revolution aimed at understanding and designing increasingly autonomous adaptive intelligent algorithms and mobile agents. In particular, it summarizes an emerging neural architecture that is capable of visually searching and navigating an unfamiliar environment while it autonomously learns to recognize, plan, and efficiently navigate toward and acquire valued goal objects. This article accordingly reviews, and outlines how to extend, the SOVEREIGN architecture of Gnadt and Grossberg (2008) (Figure 1A). The purpose of that architecture is described in the subtitle of the article: *An autonomous neural system for incrementally learning planned action sequences to navigate towards a rewarded goal*.

The architecture was called SOVEREIGN because it describes how Self-Organizing, Vision, Expectation, Recognition, Emotion, Intelligent, and Goal-oriented Navigation processes interact during adaptive mobile behaviors. The term *Self-Organizing* emphasizes that SOVEREIGN’s learning is carried out autonomously and incrementally in real time, using unconstrained combinations of unsupervised or supervised learning. *Expectation* refers to the fact that key learning processes in SOVEREIGN learn expectations that match incoming data, or predict future outcomes. Good enough matches focus attention upon expected combinations of critical features, while mismatches drive memory searches to learn better representations of an environment. *Recognition* acknowledges that SOVEREIGN learns object categories, or “chunks,” whereby to recognize objects and events. *Emotion* denotes that SOVEREIGN carries out reinforcement learning whereby unfamiliar objects can learn to become conditioned reinforcers, as well as sources of incentive motivation that can maintain attention upon valued goals, while actions to acquire those goals are carried out. Reinforcement learning also supports the learning of value categories that can recognize valued combinations of homeostatic drive inputs. *Intelligent* means that SOVEREIGN includes processes whereby sequences, or lists, of objects and positions may be temporarily stored in cognitive and spatial working memories as they are experienced in real time. Stored sequences trigger learning of sequence categories or plans, also called *list chunks*, that recognize particular sequential contexts and learn to predict the most likely future outcomes as they are modulated by reinforcement learning and incentive motivational learning. *Goal-oriented navigation* means that SOVEREIGN includes circuits for controlling exploratory and planned movements while navigating unfamiliar and familiar environments.

### 1.1. Learning Routes as a Labeled Graph of Angles Turned and Distances Traveled

**FIGURE 2:**
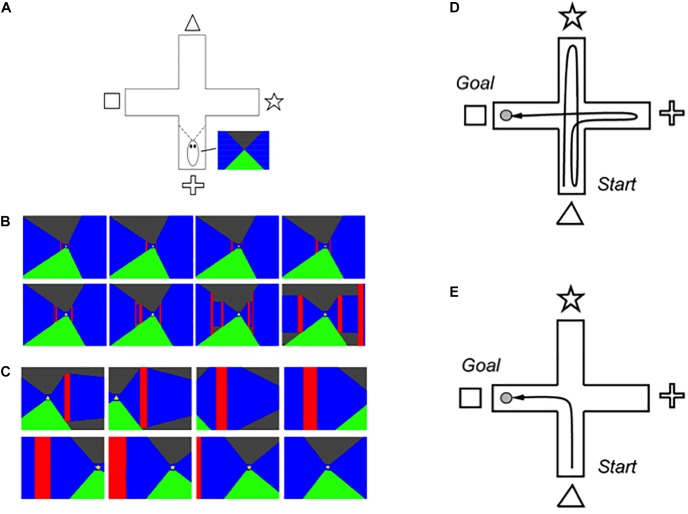
**(A)** The 3D graphical simulation of the virtual reality plus maze generates perspective views from any position within the maze. **(B)** Snapshots from the 3D virtual reality simulation depict changes in the scene during reactive homing toward the triangle cue. **(C)** During reactive approach to the triangle cue, visual motion signals trigger a reactive head orienting movement to bring the star cue into view. Two overhead views of a plus maze show **(D)** a typical initial exploratory reactive path, and **(E)** an efficient learned planned path to the goal [Adapted with permission from Gnadt and Grossberg (2008)].

SOVEREIGN used these capabilities to simulate how an animal, or animat, can autonomously learn to reach valued goal objects through planned sequences of navigational movements within a virtual reality environment. Learning was simulated in a cross maze (Figure 2A) that was seen by the animat as a virtual reality 3D rendering of the maze as it navigated it through time. At the end of each corridor in the maze, a different visual cue was displayed (triangle, star, cross, and square). Sequences of virtual reality views on two navigational routes, shown in color for vividness, are summarized in Figure 2B,C, where the floor is green, the walls are blue, the ceiling in black, and the interior corners where pairs of maze corridors meet are in red. Figure 2B illustrates how the views change as the animat navigates straight down one corridor, and Figure 2C illustrates how the views change as the animal makes a turn from facing one corridor to facing a perpendicular one.

SOVEREIGN incrementally learned how to navigate to a rewarded goal object in this cross maze, which is the perhaps the simplest environment that requires all of the SOVEREIGN designs to explore an unfamiliar visual environment (Figure 2D) while learning efficient routes whereby to acquire a valued goal, rather than less efficient or valued routes (Figure 2E). Several different types of neural circuits, systems, and learning are needed to achieve this competence. They will be described in the subsequent sections. The same mechanisms generalize to much more complex visual environments, especially because, as will be described below, all the perceptual, cognitive, and affective learning mechanisms scale to more complex environments and dynamically self-stabilize their memories using learned expectation and attention mechanisms, while the spatial and motor mechanisms are platform independent.

One key SOVEREIGN accomplishment is worthy of mention now because it illustrates how SOVEREIGN goes beyond reactive navigation to autonomously learn the most efficient routes whereby to acquire a valued goal, while rejecting less efficient routes that were taken early in the exploratory process. SOVEREIGN explains how arbitrary navigational trajectories can be incrementally learned as sequences of turns and linear movements until the next turn. In other words, the model explains how route-based navigation can learn a *labeled graph* of *angles* turned and *distances* that are traveled between turns. The angular and linear velocity signals that are experienced at such times are used in the model to learn the angles that a navigator turns, and the distances that are traveled in a straight path before the next turn.

The prediction that a labeled graph is learned during route navigation has recently received strong experimental support in Warren et al. (2017) who show how, when humans navigate in a virtual reality environment, such a labeled graph controls their navigational choices during route finding, novel detours, and shortcuts.

### 1.2. From SOVEREIGN to SOVEREIGN2: New Processes and Capabilities

SOVEREIGN did not include various brain processes and psychological functions of humans that are needed to realize a more sophisticated level of autonomous adaptive intelligence. This article summarizes some of the neural models that have been developed to explain these functions, and that can be consistently incorporated into an enhanced architecture called SOVEREIGN2. These processes have been rigorously modeled and parametrically simulated over a 40-year period, culminating in recent syntheses such as Grossberg (2013, 2017, 2018). They are reviewed heuristically here to bring together in one place the basic design principles, mechanisms, and architectures that they embody. Rigorous embodiment of all of these competences in SOVEREIGN2 will require a sustained research program. The current article provides a roadmap for that task.

The most important new perceptual, cognitive, and navigational properties emerge within feedback networks that regulate one or another kind of attention as part of resonant brain states that support conscious experiences of seeing, feeling, and knowing. These resonant states are modeled as part of Adaptive Resonance Theory, or ART. Table 1a also lists resonances that arise during auditory processing. Auditory processing will not be considered below, but is described with the others in Grossberg (2017). SOVEREIGN2 will embody such resonant dynamics, including states that in humans support consciousness, because of a deep computational connection that has been modeled between conscious states and the choice of effective task-relevant actions. ART hereby provides explanations of *what* goes on in each of our brains when we consciously see, hear, feel, or know something; *where* it is going on; and *why* evolution may have been driven to discover conscious states of mind.

**Table 1:** **(a)** Types of resonances and the conscious experiences that they embody. **(b)** Complementary What and Where cortical stream properties.

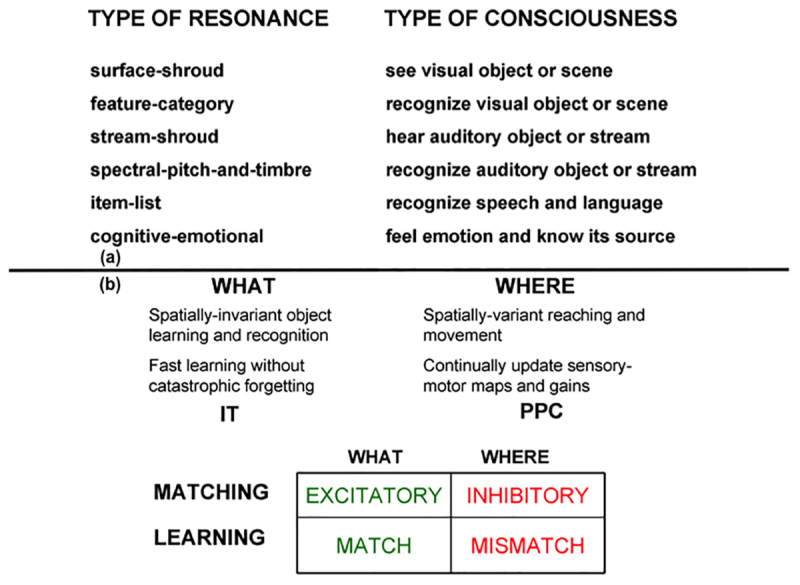


*Cortical What stream perceptual and cognitive representations can solve the stability-plasticity dilemma, using brain regions like inferotemporal (IT) cortex, where recognition categories are learned. These processes carry out excitatory matching and match-based learning. Cortical Where stream spatial and motor processes do not solve the stability-plasticity dilemma, but rather adapt to changing bodily parameters, using brain regions like posterior parietal cortex (PPC). Whereas the recognition categories in the cortical What stream become increasingly invariant at higher cortical levels with respect to object views, positions, and sizes, the cortical Where stream elaborates spatial representations of object positions and mechanisms whereby to act upon them. Together the two streams can learn to recognize and become conscious of valued objects and scenes, while directing appropriate actions toward them [Reprinted with permission from Grossberg (2017)].*

Additional processes in SOVEREIGN2 include circuits for target tracking with smooth pursuit and saccadic eye or camera movements (see section 3.2); visual form and motion perception in response to noisy and incomplete sensor signals (see section 4.13); incremental unsupervised view-, size-, and position-specific object category learning and hypothesis testing in real time in response to arbitrarily large non-stationary databases that may include unexpected events (see sections 4.2–4.9, 6.2, and 6.3); incremental unsupervised learning of view-, size-, and position- invariant object categories during free scanning of a scene with eye or camera movements (see sections 4.1, 6.1, and 6.4); selective storage in working memory of task-relevant object, spatial, or motor event sequences (see sections 4.10, 6.9, 6.10, and 7); unsupervised learning of cognitive and motor plans based upon working memory storage of event sequences in real time, and Where’s Waldo search for currently valued goal objects (see sections 6.10 and 7); unsupervised learning of reaching behaviors that automatically supports accurate tool manipulation in space (see section 5.4); unsupervised learning of present position in space using path integration during spatial navigation (see sections 6.11 and 8); platform-independent navigational control using either leg or wheel movements (see section 5.6); unsupervised learning of adaptively timed actions and maintenance of motivated attention while these actions are executed (see sections 6.7 and 6.8); and social cognitive capabilities like joint attention and imitation learning whereby a classroom of robots can learn spatial skills by each observing a teacher from its own unique spatial perspective (see section 5.5).

## 2. Brains Assemble Equations and Microcircuits Into Modal Architectures: Contrast Deep Learning

ART architectures embody key design principles that are found in advanced brains, and which enable general-purpose autonomous adaptive intelligence to work. These designs have enabled biological neural networks to offer unified principled explanations of large psychological and neurobiological databases (e.g., see Grossberg, 2013, 2017, 2018) using just a small set of mathematical *laws* or equations-such as the laws for short-term memory or STM, medium-term memory or MTM, and long-term memory or LTM-and a somewhat larger set of characteristic *microcircuits* that embody useful combinations of functional properties-such as properties of cognitive and cognitive-emotional learning and memory, decision-making, prediction, and action. Just as in physics, only a few basic equations are used to explain and predict many facts about mind and brain, when they are embodied in a somewhat larger number of microcircuits that may be thought of as the “atoms” or “molecules” of intelligence. Specializations of these laws and microcircuits are then combined into larger systems that are called *modal architectures*, where the word “modal” stands for different modalities of intelligence, such as vision, speech, cognition, emotion, and action. Modal architectures are less general than a general-purpose von Neumann computer, but far more general than a traditional algorithm from AI.

As I will illustrate throughout this article, these designs embody computational paradigms that are called *complementary computing, hierarchical resolution of uncertainty*, and *adaptive resonance*. In addition, the paradigm of *laminar computing* shows how these designs may be realized in the layered circuits of the cerebral cortex and, in so doing, achieve even more powerful computational capabilities. These computational paradigms differ qualitatively from currently popular algorithms in AI and machine learning, notably Deep Learning (Hinton et al., 2012; LeCun et al., 2015) and its variants like Deep Reinforcement Learning (Mnih et al., 2013). Despite their successes in demonstrating various recent applications, these algorithms do not come close to matching the generality, adaptability, and intelligence that is found in models that more closely emulate brain designs. As just one of many problems, Deep Learning algorithms are susceptible to undergoing catastrophic forgetting, or an unexpected collapse of the memory of previously learned information while new information is being learned, a property that is shared by all variants of the classical back propagation algorithm (Grossberg, 1988). This kind of problem becomes increasingly destructive as a Deep Learning algorithm tries to learn from very large databases. The ART-based systems that are summarized below do not experience these problems.

No less problematic is that Deep Learning is just a feedforward adaptive filter. It does not carry out any of the basic kinds of information processing that are typically identified as “intelligent,” but which are carried out within ART and other biological learning algorithms that are embedded within neural network architectures. Deep Learning has none of the architectural features, such as learned top-down expectations, attentional focusing, and mismatch-mediated memory search and hypothesis testing, that are needed for stable learning in a non-stationary world of Big Data.

Perhaps these problems are why Geoffrey Hinton said in an *Axios* interview on September 15, 2017 (LeVine, 2017) that he is “deeply suspicious of back propagation…I don’t think it’s how the brain works. We clearly don’t need all the *labeled data*…My view is, *throw it all away and start over*” (italics mine). This essay illustrates that we do not need to start over.

Section 17 in Grossberg (1988) lists 17 different learning and performance properties of Back Propagation and Adaptive Resonance Theory. The third of the 17 differences between Back Propagation and ART is that ART does not need labeled data to learn. ART can learn using arbitrary combinations of unsupervised and supervised learning. ART also does not experience any of the computational problems that compromise Back Propagation and Deep Learning, including catastrophic forgetting.

## 3. Building Upon Three Basic Design Themes: Balancing Reactive and Planned Behaviors

The original SOVEREIGN architecture contributed models of three basic design themes about how advanced brains work. The first theme concerns how brains learn to balance between reactive and planned behaviors. During initial exploration of a novel environment, many reactive movements may occur in response to unfamiliar and unexpected environmental cues (Leonard and McNaughton, 1990). These movements may seem initially to be random, as an animal orients toward and approaches many stimuli (Figure 2D). As the animal becomes familiar with its surroundings, it learns to discriminate between objects likely to yield a reward and those that lead to punishment or to no valued consequences. Such approach-avoidance behavior is typically learned via reinforcement learning during a perception-cognition-emotion-action cycle in which an action and its consequences elicit sensory cues that are associated with them. When objects are out of direct viewing or reaching ranges, reactive exploratory movements may be triggered to bring them closer. Eventually, reactive exploratory behaviors are replaced by more efficient planned sequential trajectories within a familiar environment (Figure 2E). One of the main goals of SOVEREIGN was to explain how erratic reactive exploratory behaviors trigger learning to carry out organized planned behaviors, and how both reactive and planned behaviors may remain balanced so that planned behaviors can be carried out where appropriate, without losing the ability to respond quickly to novel reactive challenges.

### 3.1. Parallel Streams for Computing Visual Form and Motion

**FIGURE 3:**
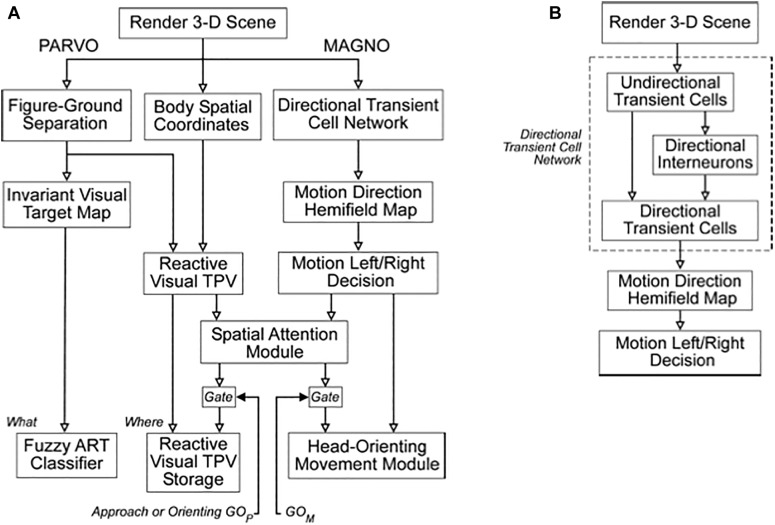
**(A)** The Visual Form System (PARVO) and Motion System (MAGNO) flow diagrams depict the stages of visual processing in SOVEREIGN. **(B)** Detailed stages of motion processing within the Motion System are shown in this diagram. The Directional Transient Cell Network module comprises multiple stages of processing. The Motion Left/Right Decision generates signals that are capable of eliciting a reactive left or right head-orienting signal. The several transient cell stages enable the Motion System to retain its directional selectivity in response to motions at variable speeds. See the text for details.

One way that SOVEREIGN realizes a flexible balance between reactive and planned behaviors is its organization into parallel streams for computing visual form and motion. In Figure 3A, these streams are labeled PARVO and MAGNO, corresponding to contributions at early visual processing stages of parvocellular cells to form processing and magnocellular cells to motion processing (e.g., Maunsell and Newsome, 1987; DeYoe and Van Essen, 1988; Maunsell et al., 1990; Schiller et al., 1990). Roughly speaking, the form stream supports sustained attention upon foveated objects, whereas the motion stream attracts attention and bodily movements in response to sudden changes, including motions, in the periphery. sections 3.2 and 4.13 will further describe how SOVEREIGN carries out form processing and will outline how SOVEREIGN2 can achieve much more powerful form processing capabilities. Figure 3B provides a more detailed summary of the early motion processing that enables SOVEREIGN to track objects moving at variable speeds (Chey et al., 1997; Berzhanskaya et al., 2007). Orienting movements to a source of motion were controlled algorithmically in SOVEREIGN; e.g., see the Head-Orienting Movement Module in Figure 3A.

### 3.2. Log Polar Retinas and Fixating Unpredictably Moving Targets With Eye Movements

Many primate retinas have a localized region of high visual acuity that is called the *fovea*, with resolution decreasing with distance from the fovea (see Supplementary Figure S4) to realize a *cortical magnification factor* whereby spatial representations of retinal inputs in the visual cortex get coarser as they move from the foveal region to the periphery (Daniel and Whitteridge, 1961; Fischer, 1973; Tootell et al., 1982; Schwartz, 1984; Polimeni et al., 2006). The cortical magnification factor is approximated by a log-polar function, which allows a huge reduction in the number of cells that are needed to see Schwartz (1984), Wallace et al. (1984), Schwartz et al. (1995). However, because of this retinal organization, eye and head movements are needed to move the fovea to look at objects of interest.

Both smooth pursuit movements and saccadic eye movements are used to keep the fovea looking at objects of interest. During a smooth pursuit movement, as the eyes track a moving target in a given direction, the entire scene moves in the opposite direction on the retina (Supplementary Figure S1). Why does not this background motion interfere with tracking by causing an involuntary motion, called nystagmus, in the opposite direction than the target is moving? How does accurate tracking continue, even after the eye catches up with the moving target, so that there is no net speed of the target on the fovea, and thus no retinal slip signals from the foveal region of the eyes to move them toward the target?

Remarkably, both of these questions seem to have the same answer, which includes the fact that the background motion facilitates tracking, rather than interfering with it, in the manner that is summarized in Supplementary Figures S1, S2. Supplementary Figure S1 summarizes the fact that, for fixed target speed, as the target speed on the retina decreases due to increasingly good target tracking, the background speed in the opposite direction on the retina increases. Supplementary Figure S2 schematizes the smooth pursuit eye movement, or SPEM, model of Pack et al. (2001) of how cells in the dorsal Medial Superior Temporal region (MSTd), which are activated by the background motion, excite cells that are sensitive to the *opposite* direction in the ventral MST (MSTv) region. The MSTv cells are the ones that control the movement commands whereby the eyes pursue the moving target. When the eyes catch up to the target, they can maintain accurate foveation even in the absence of retinal slip signals, because background motion signals compensate for the reduced retina speed of the target, and can thus be used to accurately move the eyes in the desired direction at the target speed (Supplementary Figure S1). This kind of SPEM model can replace the Head-Orienting Movement Module in SOVEREIGN if an animat with orienting eyes or cameras is used.

When a valued target suddenly changes its speed or direction of motion, then smooth pursuit movements may be insufficient. Ballistic saccadic movements can then catch up with the target. Animals such as humans and monkeys can coordinate smooth pursuit and ballistic saccadic eye movements to catch up efficiently. Indeed, the current speed and direction of smooth pursuit when the target suddenly changes its speed or direction may be used to calibrate a ballistic saccade with the best chance to catch up. This kind of predictive coordination is achieved by the SAC-SPEM model of Grossberg et al. (2012). The sheer number of brain regions that work together to accomplish such coordination (Supplementary Figure S3) will challenge future mobile robotic designers to embody this tracking competence in the simplest possible way.

## 4. Building Upon Three Basic Design Themes: Complementary Computing, Hierarchical Resolution of Uncertainty, and Adaptive Resonance

The second design theme is that advanced brains are organized into parallel processing streams with computationally complementary properties (Grossberg, 2000, 2017). Complementary computing means that each stream’s properties are related to those of a complementary stream much as a key fits into a lock, or two pieces of a puzzle fit together. The mechanisms that enable each stream to compute one set of properties prevent it from computing a complementary set of properties. As a result, each of these streams exhibits complementary strengths and weaknesses. Interactions between these processing streams use multiple processing stages to overcome their complementary deficiencies and generate psychological properties that lead to successful behaviors. This interactive multi-stage process is called *hierarchical resolution of uncertainty*.

Two of these complementary streams are the ventral What cortical stream for object perception and recognition, and the dorsal Where (or Where/How) cortical processing stream for spatial representation and action (Ungerleider and Mishkin, 1982; Mishkin, 1982; Mishkin et al., 1983; Goodale et al., 1991; Goodale and Milner, 1992). Key properties of these cortical processing streams have been shown to be computationally complementary (Table 1b).

### 4.1. Invariant Object Category Learning

One of several basic reasons for this particular kind of complementarity is that the cortical What stream learns object recognition categories that become substantially invariant under changes in an object’s view, size, and position at higher cortical processing stages, such as at the anterior inferotemporal cortex (ITa) and beyond (Tanaka, 1997, 2000; Booth and Rolls, 1998; Fazl et al., 2009; Cao et al., 2011; Chang et al., 2014). These invariant object categories have a compact representation that enables valued objects to be recognized without causing the combinatorial explosion that would have occurred if our brains needed to store every individual exemplar of every object that was ever experienced. However, because they are invariant, these categories cannot, by themselves, locate and act upon a desired object in space. Cortical Where stream spatial and motor representations can locate objects and trigger actions toward them, but cannot recognize them. By interacting together, the What and Where streams can consciously see and recognize valued objects *and* direct appropriate goal-oriented actions toward them.

**FIGURE 4:**
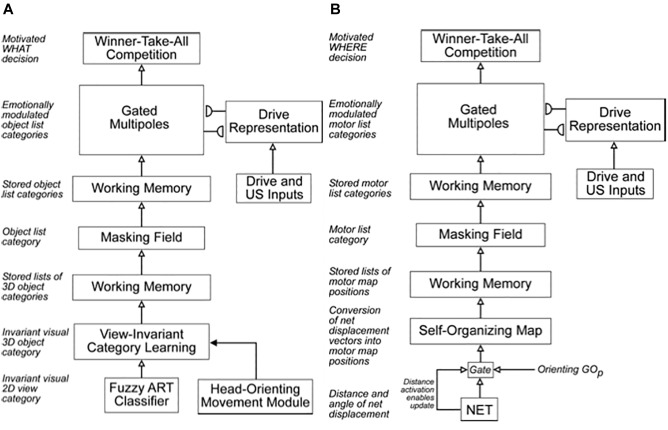
**(A)** The Visual Working Memory and Planning System computes motivationally reinforced representations of sequences of 3D object categories. **(B)** The Motor Working Memory and Planning System computes motivationally reinforced representations of sequences of motor map positions/directions. See the text for details. [Reprinted with permission from Gnadt and Grossberg (2008)].

The original SOVEREIGN model explained simple properties of how such invariant categories are learned as an animal, or animat, explores a novel environment. It used log-polar preprocessing of input images, followed by coarse-coding and algorithmic shift operations, to generate size-invariant and position-invariant input images. These preprocessed images were then input to a Fuzzy ART classifier (Carpenter et al., 1991b) for learning invariant visual 2D view-specific categories whereby SOVEREIGN could recognize an object at variable distances. These view-specific categories were converted into categories that were view-invariant, as well as positionally invariant and size-invariant, by algorithmically associating multiple view-specific categories with a shared view-invariant category (Figure 4A).

Since SOVEREIGN was published, the 3D ARTSCAN SEARCH model was developed to explain how humans and other primates may accomplish incremental unsupervised learning of view-, position-, and size-invariant categories, without any algorithmic shortcuts, and how these invariant categories can be used to trigger a cognitively or motivationally driven Where’s Waldo search for a desired object in a cluttered scene (Fazl et al., 2009; Grossberg, 2009b; Cao et al., 2011; Foley et al., 2012; Chang et al., 2014; Grossberg et al., 2014). These important *Recognition* and Where’s Waldo search capabilities, which will be further discussed in sections 6.1 and 6.4, can also be incorporated into SOVEREIGN2 instead of the bottom two category learning processes in Figure 4A.

### 4.2. Adaptive Resonance Theory: A Universal Design for Autonomous Classification and Prediction

The ART in the Fuzzy ART algorithm abbreviates Adaptive Resonance Theory, which was introduced in 1976 (Grossberg, 1976a,b) and developed into the most advanced cognitive and neural theory of how advanced brains learn to attend, recognize, and predict objects and events in complex changing environments that may be filled with unexpected events. ART currently has an unrivalled explanatory and predictive range about how processes of consciousness, learning, expectation, attention, resonance, and synchrony interact in advanced brains. Along the way, all of the foundational hypotheses of ART have been confirmed by later psychological and neurobiological experiments. See Grossberg (2013, 2017, 2018) for recent reviews and syntheses.

ART’s significance is highlighted by the fact that its design principles and mechanisms can be derived from a *thought experiment* whose simple assumptions are familiar to us all as facts that we experience ubiquitously in our daily lives. These facts embody environmental constraints which, taken together, define a multiple constraint problem that evolution has solved in order to enable humans and other higher animals to be able to autonomously learn to attend, recognize, and predict their unique and changing worlds. Such a competence is essential in autonomous adaptive mobile agents, which is why some ART algorithms were already algorithmically implemented in SOVEREIGN.

### 4.3. Predictive Brain: Intention, Attention, and Resonance Solve the Stability-Plasticity Dilemma

One of the critical properties of ART that enable it to support open-ended incremental autonomous learning is that resonant states can trigger rapid learning about a changing world while solving the *stability-plasticity dilemma*. This dilemma asks how can our brains learn quickly without being forced to forget previously learned, but still useful, memories just as quickly?

The stability-plasticity dilemma was articulated before the *catastrophic forgetting* problem was stated (French, 1999), and clarifies that it is a problem of *balance* between fast learning and stable memory. Catastrophic forgetting means that an unpredictable part of previously learned memories can rapidly and unpredictably collapse during new learning. This problem becomes particularly acute when learning any kind of Big Data problem, notably during the kind of open-ended incremental learning that an autonomous adaptive robot might need to do as it navigates unfamiliar environments. A catastrophic collapse of previous memories while trying to completely learn about a huge database, not to mention a database that is continually changing through time, is intolerable in any application that can have serious real world consequences. Popular machine learning algorithms such as Back Propagation and its recent variant, Deep Learning (Hinton et al., 2012; LeCun et al., 2015), do not solve the catastrophic forgetting problem. In brief, Deep Learning is unreliable.

A resonant brain state is a dynamical state during which neuronal firings across a brain network are amplified and synchronized when they interact via reciprocal excitatory feedback signals during an attentive matching process that occurs between bottom-up and top-down pathways. In the case of learning recognition categories, the bottom-up pathways are adaptive filters that tune their adaptive weights, or LTM traces, to more reliably activate the category that best matches the input feature patterns that activate them. The top-down pathways are learned recognition expectations whose LTM traces focus attention upon a prototype of critical features that best predict the active category. As will be explained in greater detail below (see Figure 7 below), a resonance of this kind is called a *feature-category resonance* in order to distinguish it from the multiple other kinds of resonances that dynamically stabilize learning in different brain systems.

A resonance represents a system-wide consensus that the attended information is worthy of being learned. It is because resonances can trigger fast learning that they are called *adaptive* resonances, and why the theory that explicates them is called Adaptive Resonance Theory. ART’s proposed solution of the stability-plasticity dilemma mechanistically links the process of stable learning and memory with the mechanisms of Consciousness, Expectation, Attention, Resonance, and Synchrony that enable it. Due to their mechanistic linkage, these processes are often abbreviated as the CLEARS processes.

ART hereby predicts that interactions among CLEARS mechanisms solve the stability-plasticity dilemma. That is why humans and other higher animals are *intentional* and *attentional* beings who use learned expectations to pay attention to salient objects and events, why “all conscious states are resonant states,” and how brains can learn both *many-to-one maps* (representations whereby many object views, positions, and sizes learn to activate the same invariant object category), and *one-to-many maps* (learned representations that enable us to expertly know many things about individual objects and events).

As will be explained in greater detail below, the link between Consciousness, Learning, and Resonance is a particularly important one for understanding both characteristically human experiences and how future machine learning algorithms may embody them.

### 4.4. Object Attention Dynamically Stabilizes Learning Using the ART Matching Rule

ART solves the stability-plasticity dilemma by using learned expectations and attentional focusing to *selectively* process only those data that are predicted to be relevant in any given situation. Because of the CLEARS relationships, such selective attentive processing also solves the stability-plasticity dilemma.

For this to work, the correct laws of object attention need to be used. ART has predicted how object attention is realized in human and other advanced primate brains (e.g., Grossberg, 1980, 2013; Carpenter and Grossberg, 1987a, 1991). In order to dynamically stabilize learning, the learned expectations that focus attention obey a *top-down, modulatory on-center, off-surround* network. This network is said to obey the ART Matching Rule.

In such a network, when a bottom-up input pattern is received at a processing stage, it can activate its target cells, if nothing else is happening. When a top-down expectation pattern is received at this stage, it can provide excitatory modulatory, or priming, signals to cells in its on-center, and driving inhibitory signals to cells in its off-surround, if nothing else is happening. The on-center is *modulatory* because the off-surround network also inhibits the on-center cells, and these excitatory and inhibitory inputs are approximately balanced (“one-against-one”). When a bottom-up input pattern and a top-down expectation are both active, cells that receive both bottom-up excitatory inputs and top-down excitatory priming signals can fire (“two-against-one”), while other cells are inhibited. In this way, only cells can fire whose features are “expected” by the top-down expectation, and an attentional focus starts to form at these cells. As a result only attended feature patterns are learned. The system wherein category learning takes place is thus called an *attentional system.*

The property of the ART Matching Rule that bottom-up sensory activity may be enhanced when matched by top-down signals is in accord with an extensive neurophysiological literature showing the facilitatory effect of attentional feedback (e.g., Sillito et al., 1994; Luck et al., 1997; Roelfsema et al., 1998). This property contradicts popular models, such as Bayesian Explaining Away models, in which matches with top-down feedback cause only suppression (Mumford, 1992; Rao and Ballard, 1999). A related problem is that suppressive matching circuits cannot solve the stability-plasticity dilemma.

An ART expectation is a *top-down, adaptive*, and *specific* event that activates its target cells during a *match* within the attentional system. “Adaptive” means that the top-down pathways contain adaptive weights that can learn to encode a prototype of the recognition category that activates it. “Specific” means that each top-down expectation reads out its learned prototype pattern. One psychophysiological marker of such a resonant match is the processing negativity, or PN, event-related potential (Grossberg, 1978, 1984b; Näätänen, 1982; Banquet and Grossberg, 1987).

### 4.5. ART Is a Self-Organizing Production System: Lifelong Learning of Expertise

The above properties of an expectation are italicized because, as will be seen below, they are *computationally complementary* to those of an *orienting system* that enables ART to autonomously learn about arbitrarily many novel events in a non-stationary environment without experiencing catastrophic forgetting. As will be explained more fully below, if a top-down expectation mismatches an incoming bottom-up input pattern too much, the orienting system is activated and drives a memory search and hypothesis testing for either a better-matching category if the input represents information that is familiar to the network, or a novel category if it is not.

Taken together, the ART attentional and orienting systems constitute a *self-organizing production system* that can learn to become increasingly expert about the world that it experiences throughout the life span of the individual or machine into which it is embedded.

### 4.6. ART Can Carry Out Open-Ended Stable Learning of Huge Non-stationary Databases

Our ability to achieve learning throughout life can be stated in another way that emphasizes its critical importance in human societies no less than in designing autonomous adaptive robots with real intelligence: Without stable memories of past experiences, we could learn very little about the world, since our present learning would wash away previous memories unless we continually rehearsed them. But if we had to continuously rehearse everything that we learned, then we could learn very little, because there is just so much time in a day to rehearse. Having an active top-down matching mechanism greatly amplifies the amount of information that humans can quickly learn and stably remember about the world. This capability, in turn, sets the stage for developing a sense of self, which requires that we can learn and remember a record of many experiences that are uniquely ours over a period of years.

With appropriately implemented ART algorithms on board, a SOVEREIGN2 robot can continue to learn indefinitely for its entire lifespan.

### 4.7. Large-Scale Machine Learning Applications in Engineering and Technology

ART enables a general-purpose category learning, recognition, and prediction capability that has already been used in multiple large-scale applications in engineering and technology. When it is embodied completely enough in SOVEREIGN2, then SOVEREIGN2 can also be used to carry out such applications, and can do so with the advantage being able to navigate environments where these applications occur.

Fielded applications include: airplane design (including the Boeing 777); medical database diagnosis and prediction; remote sensing and geospatial mapping and classification; multidimensional data fusion; classification of data from artificial sensors with high noise and dynamic range (synthetic aperture radar, laser radar, multi-spectral infrared, night vision); speaker-normalized speech recognition; sonar classification; music analysis; automatic rule extraction and hierarchical knowledge discovery; machine vision and image understanding; mobile robot controllers; satellite remote sensing image classification; electrocardiogram wave recognition; prediction of protein secondary structure; strength prediction for concrete mixes; tool failure monitoring; chemical analysis from ultraviolet and infrared spectra; design of electromagnetic systems; face recognition; familiarity discrimination; and power transmission line fault diagnosis. Some of these applications are listed at http://techlab.bu.edu/resources/articles/C5/.

### 4.8. Mathematically Provable ART Learning Properties Support Large-Scale Applications

It is because the good learning properties of ART have been mathematically proved and tested with comparative computer simulation benchmarks that ART has been used with confidence in these applications (e.g., Carpenter and Grossberg, 1987a,b, 1990; Carpenter et al., 1989, 1991a,b, 1992, 1998).

These theorems prove how ART can rapidly learn, from arbitrary combinations of unsupervised and supervised trials, to categorize complex, and arbitrarily large, non-stationary databases, dynamically stabilize their learned memories, directly access the globally best matching categories with no search during recognition, and use these categories to predict the most likely outcomes in a given situation.

In particular, ART provably solved the catastrophic forgetting problem that other approaches to machine learning have failed to solve.

### 4.9. ART Solves the Explainable AI Problem and Extracts Knowledge Hierarchies From Data

ART offers a solution of another problem that other researchers in machine learning and AI are still seeking. The learned weights of the fuzzy ARTMAP algorithm (Carpenter et al., 1992) translate, at any stage of learning, into fuzzy IF-THEN rules that “explain” why the learned predictions work. Understanding why particular predictions are made is no less important than their predictive success in applications that have life or death consequences, such as medical database diagnosis and prediction, to which ART has been successfully applied. This problem has not yet been solved in traditional AI, as illustrated by the current DARPA Explainable AI program (XAI^1^).

In addition, ART can self-organize hierarchical knowledge structures from masses of incomplete and partially incompatible data taken from multiple observers who do not communicate with each other, and who may use different combinations of object names and sensors to incrementally collect their data at different times, locations, and scales (Carpenter et al., 2005; Carpenter and Ravindran, 2008). If swarms of SOVEREIGN2 robots collect data in this distributed way, then they can share it wirelessly to self-organize such cognitive hierarchies of rules.

### 4.10. Cognitive and Spatial Working Memories and Plans

Figure 4A also summarizes higher cognitive and cognitive-emotional processes that are modeled in SOVEREIGN. Together with Figure 4B, these contribute to SOVEREIGN’s *Intelligent* and *Goal-oriented navigation* processing whereby cognitive working memories (Figure 4A) and spatial working memories (Figure 4B) provide the information whereby cognitive plans (Figure 4A) and spatial plans (Figure 4B) are learned and used to control actions to acquire valued goals. The cognitive working memory temporarily stores the temporal order of sequences of invariant object categories that represent recently experienced objects. These sequences are themselves categorized during learning of cognitive plans, or *list chunks*, that fire selectively in response to particular stored object sequences. Such a network of list chunks is called a Masking Field (Grossberg, 1978; Cohen and Grossberg, 1986, 1987; Grossberg and Myers, 2000; Grossberg and Kazerounian, 2011; Kazerounian and Grossberg, 2014). The corresponding spatial working memory and Masking Field in Figure 4B do the same thing for the stored sequences of navigational movements—notably combinations of turns and straight excursions in space—that SOVEREIGN carries out while exploring the maze. These processes will be discussed further in sections 6.9, 6.10, and 7, notably how they need to be enhanced in SOVEREIGN2 to achieve selective processing and storage of only task-relevant sequences of information.

### 4.11. Reinforcement Learning and Incentive Motivation to Acquire Valued Goals

These cognitive and spatial processes do not themselves compute indices of predictive success and failure. The processes that accomplish goal-oriented selectivity—including gated multipoles and drive representations—occur next (See Figure 12 below). These reinforcement learning and incentive motivational processes enable SOVEREIGN to select, amplify, and sustain in working memory those previous event sequences that have led to predictive success in the past, and to use these list categories to predict the actions most likely to achieve valued goals in the future. These processes will be further discussed in sections 6.5–6.7.

### 4.12. Prefrontal Regulation of Cognitive and Cognitive-Emotional Dynamics

**FIGURE 5:**
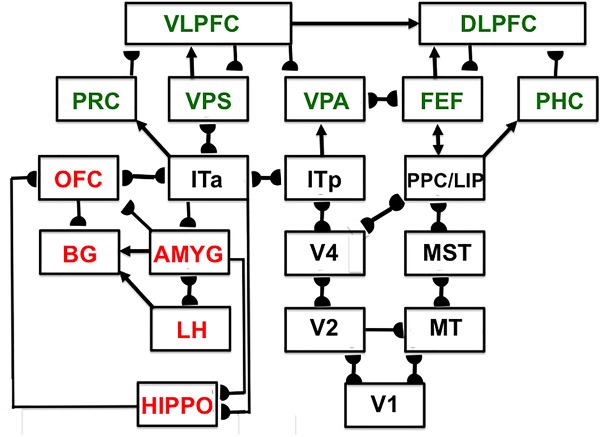
Macrocircuit of the main brain regions, and connections between them, that are modeled in the *predictive Adaptive Resonance Theory* (pART) model of working memory and cognitive-emotional dynamics. Abbreviations in green denote brain regions used in working memory dynamics, whereas abbreviations in red denote brain regions used in cognitive-emotional dynamics. Black abbreviations refer to brain regions that process visual data during visual perception and are used to learn visual object categories. Arrows denote non-adaptive excitatory synapses. Hemidiscs denote adaptive excitatory synapses. Many adaptive synapses are bidirectional, thereby supporting synchronous resonant dynamics among multiple cortical regions. The output signals from the basal ganglia that regulate reinforcement learning and gating of multiple cortical areas are not shown. Also not shown are output signals from cortical areas to motor responses. V1, striate, or primary, visual cortex; V2 and V4, areas of prestriate visual cortex; MT, middle temporal cortex; MST, medial superior temporal area; ITp, posterior inferotemporal cortex; ITa, anterior inferotemporal cortex; PPC, posterior parietal cortex; LIP, lateral intraparietal area; VPA, ventral prearcuate gyrus; FEF, frontal eye fields; PHC, parahippocampal cortex; DLPFC, dorsolateral hippocampal cortex; HIPPO, hippocampus; LH, lateral hypothalamus; BG, basal ganglia; AMGY, amygdala; OFC, orbitofrontal cortex; PRC, perirhinal cortex; VPS, ventral bank of the principal sulcus; VLPFC, ventrolateral prefrontal cortex. See text for further details. [Reprinted with permission from Grossberg (2018)].

Since SOVEREIGN appeared, the predictive Adaptive Resonance Theory, or pART, model (Grossberg, 2018) has proposed how several parts of the prefrontal cortex (PFC) learn to interact with multiple brain regions to carry out cognitive and spatial working memory, planning, and cognitive-emotional processes. The seven prefrontal cortical regions marked in green in Figure 5 illustrate this complexity. As one of its several explanatory accomplishments, pART clarifies how a top-down cognitive prime from the PFC can bias object attention in the What cortical stream to anticipate expected objects and events, while it also focuses spatial attention in the Where cortical stream to trigger actions that acquire currently valued objects (Fuster, 1973; Baldauf and Desimone, 2014; Bichot et al., 2015). Section 7 will summarize several of these enhanced capabilities of pART. As these enhanced capabilities of pART are incorporated into SOVEREIGN2, it will be able to carry out more sophisticated cognitive, cognitive-emotional, and Where’s Waldo search capabilities than can the SOVEREIGN or the 3D ARTSCAN SEARCH models.

The pART model embodies several different kinds of brain resonances. In particular, the Fuzzy ART classifier in Figure 4A is an algorithmic realization of the kind of *feature-category resonance* that links cortical areas V4 and ITp in Figure 5. Such a resonance focuses attention upon salient combinations of features while it triggers learning in the bottom-up adaptive filters and top-down learned expectations that bind the attended feature patterns to the object categories that are used to recognize them. Adaptive Resonance Theory, or ART, explicates several different kinds of brain resonances and their different functional roles, as will be further discussed in sections 4.15 and 4.16.

### 4.13. From Incomplete Early Sensory Representations to Conscious Awareness and Effective Action

Hierarchical resolution of uncertainty occurs even at the earliest cortical processing levels. One of the most important consequences of hierarchical resolution of uncertainty arises from the fact that the perceptual representations that are computed at early processing stages may not be able to control effective actions. These processing stages did not have to be included in SOVEREIGN because it directly processed simplified virtual reality images (Figure 2). SOVEREIGN thus did not have to deal with problems that are raised when images are processed by noisy detectors that are made from biological or physical components.

For example, visual images that are registered on the retina of a human eye are noisy and incomplete due to the existence of the blind spot and retinal veins, which prevent visual features from being registered on the retina at their positions (Supplementary Figure S4). Supplementary Figure S5 illustrates this problem with the simple example of a line that is occluded by the blind spot and some retinal veins. The parts of the line that are occluded need to be completed at higher processing stages before actions like looking and reaching can be directed to these positions. Processes of boundary completion and surface filling-in are needed to generate a sufficiently complete, context-sensitive, and stable visual surface representation upon which subsequent actions can be based (Grossberg, 1994, 1997, 2013, 2017).

**FIGURE 6:**
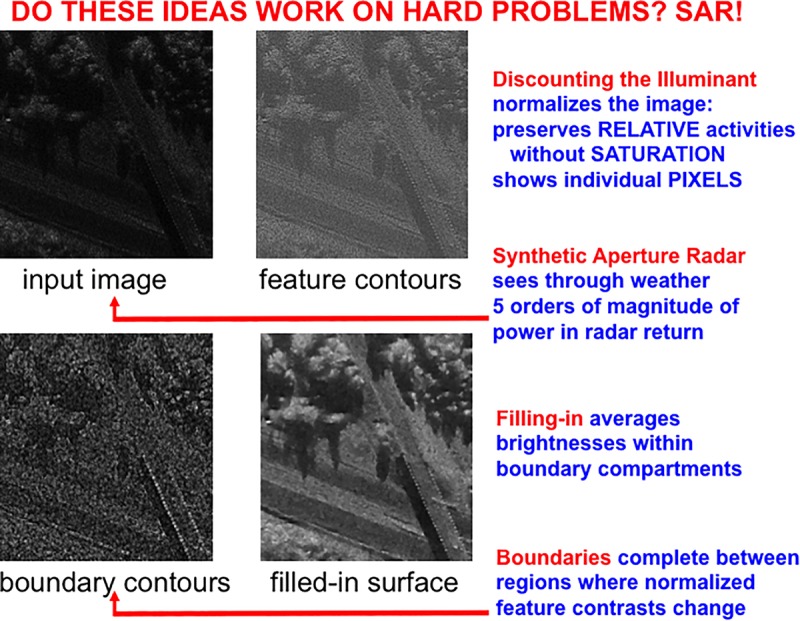
A Synthetic Aperture Radar (SAR) input image (upper left panel) is normalized (upper right panel) before it is used to compute boundaries (lower left panel) that join statistically regular pixel contrasts. Then the still highly pixelated normalized image fills-in the compartments defined by the boundaries (lower right image) to generate a representation of a scene in upper New York State in which a diagonal road crosses over a highway in a wooded area. See text for details. [Adapted with permission from Mingolla et al. (1999)].

The front end of SOVEREIGN2 can be consistently extended to include these boundary completion and surface filling-in processes, instead of the Render 3-D Scene and Figure-Ground Separation processes in Figure 3A. SOVEREIGN2 can then function even using sensory detectors that may be pixelated or degraded in various ways due to use. Such detectors include artificial sensors such as Synthetic Aperture Radar, Laser Radar, and Multispectral Infrared. Synthetic Aperture Radar, or SAR, can be used to process images that can see through the weather. Figure 6 shows a computer simulation of how a SAR image can be processed by boundary completion and surface filling-in processes that compensate for sensor failures.

Boundary completion and surface filling-in processes illustrate one of the best known examples of complementary computing (Grossberg, 1984a, 1994, 1997; Grossberg and Mingolla, 1985): Boundaries are completed *inwardly* between pairs or greater numbers of inducers in an *oriented* fashion. Boundary completion is also triggered after the processing stage where cortical complex cells pool signals from simple cells that are sensitive to opposite contrast polarities, thus becoming *insensitive* to direction of contrast. Because they pool over opposite contrast polarities-including achromatic black–white contrasts, and chromatic red–green and blue–yellow contrasts-boundaries cannot represent conscious visual qualia. That is, *all boundaries are invisible.* Surface filling-in of brightness and color spread *outwardly* in an *unoriented* fashion until they hit a boundary, or attenuate due to their spatial spread. Surface filling-in is also *sensitive* to direction of contrast. *All conscious percepts of visual qualia are surface percepts*. These three pairs of properties (inward vs. outward, oriented vs. unoriented, and insensitive vs. sensitive to direction of contrast) are manifestly complementary.

### 4.14. Why Did Evolution Discover Consciousness? Conscious States Control Adaptive Actions

The above review of some of the early processing stages in the visual system provides a foundation for understanding how ART provides a rigorous computational proposal both for *what* happens in each brain and *how* and *where* it happens as it learns to consciously see, hear, feel, or know something, as well as for *why* evolution was driven to discover conscious states in the first place (Grossberg, 2017). In particular, as noted above, in order to resolve the computational uncertainties caused by complementary computing, the brain needs to use multiple processing stages that include interactions between pairs of complementary cortical processing streams to realize a *hierarchical resolution of uncertainty.*

Because the light that falls on our retinas may be occluded by the blind spot, multiple retinal veins, and all the other retinal layers through which light passes before it reaches the light-sensitive photoreceptors (Supplementary Figures S4, S5), these retinal images are highly noisy and incomplete. Using them to control actions like looking and reaching could lead to incorrect, and potentially disastrous, actions.

In order to compute the functional units of visual perception, namely 3D boundaries and surfaces, three pairs of computationally complementary uncertainties need to be resolved using a hierarchical resolution of uncertainty. If this is indeed the case, then why do not the earlier processing stages undermine behavior by causing incorrect, and potentially disastrous, actions to be taken? In the case of visual perception, the proposed answer is that *brain resonance, and with it conscious awareness of visual qualia, is triggered at the cortical processing stage that represents 3D surface representations, after they are complete, context-sensitive, and stable enough to control visually based actions like attentive looking and reaching*. The conscious state is an “extra degree of freedom” that selectively “lights up” this surface representation and enables our brains to selectively use it to control adaptive actions.

ART hereby links the evolution of consciousness to the ability of advanced brains to learn how to control adaptive actions. In the case of visual perception, this surface representation is predicted to occur in prestriate visual cortical area V4, where a *surface-shroud resonance* that supports conscious seeing is predicted to be triggered between V4 and the posterior parietal cortex, or PPC (Figure 5), before it propagates both top-down to V2 and V1 and bottom-up to the PFC. The PPC is in the dorsal Where cortical stream. An *attentional shroud* is spatial attention that fits itself to the shape of an attended object surface (Tyler and Kontsevich, 1995). An active surface-shroud resonance maintains spatial attention on the surface throughout the duration of the resonance. When spatial attention shifts, the resonance collapses and another object can be attended.

While a surface-shroud resonance is still active, it regulates saccadic eye movement sequences that foveate salient features on the attended object surface. These properties mechanistically explain the distinction between two different functional roles of PPC: its control of top-down *attention* from PPC to V4 and its control of the *intention* to move, a distinction that has been reported in both psychophysical and neurophysiological experiments (e.g., Andersen et al., 1985; Gnadt and Andersen, 1988; Snyder et al., 1997, 1998). How spatial attention regulates the learning of invariant object categories during free scanning of a scene using its intentional choice of scanning eye movements that foveate sequences of salient surface features will be summarized in section 6.4.

The proposed link between consciousness and action is relevant to the design of future autonomous adaptive robots, and provides a new computational perspective for discussing whether machine consciousness is possible, and how it may be necessary to control a robot’s choice of context-appropriate actions.

### 4.15. Synchronized Resonances for Seeing and Knowing: Visual Neglect and Agnosia

Many psychological and neurobiological data have been explained using ART resonances. For example, surface-shroud resonances for conscious seeing and feature-category resonances for conscious knowing of visual events can synchronize via shared visual representations in the prestriate cortical areas V2 and V4 when a person sees and knows about a familiar object (Figure 5). A lesion of the parietal cortex in one hemisphere can prevent a surface-shroud resonance from forming, thereby leading to the clinical syndrome of *visual neglect*, whereby an individual may draw only one half of the world, dress only one half of the body, and make erroneous reaches. A lesion of the inferotemporal cortex can prevent a feature-category resonance from forming, thereby leading to the clinical syndrome of *visual agnosia*, whereby a human can accurately reach for an object without knowing anything about it. See Grossberg (2017) for mechanistic explanations.

### 4.16. Classification of Adaptive Resonances for Seeing, Hearing, Feeling, Knowing, and Acting

In addition to the surface-shroud resonances that supports conscious seeing and the feature-category resonances that support conscious knowing, ART explains what resonances support hearing and feeling, and how resonances supporting knowing are synchronously linked to them. All of these resonances support different kinds of learning that solve the stability-plasticity dilemma; e.g., visual and auditory learning, reinforcement learning, cognitive recognition learning, and cognitive speech and language learning.

In summary, *surface-shroud resonances* support conscious percepts of visual qualia. *Feature-category resonances* support conscious learning and recognition of visual objects and scenes. Both kinds of resonances may synchronize during conscious seeing and recognition, so that we know what a familiar object is as we see it. *Stream-shroud resonances* support conscious percepts of auditory qualia. *Spectral-pitch-and-timbre resonances* support conscious learning and recognition of sources in auditory streams. Stream-shroud and spectral-pitch-and-timbre resonances may synchronize during conscious hearing and recognition of auditory streams. *Item-list resonances* support conscious learning and recognition of speech and language. They may synchronize with stream-shroud and spectral-pitch-and-timbre resonances during conscious hearing of speech and language, and build upon the selection of auditory sources by spectral-pitch-and-timbre resonances in order to recognize the acoustical signals that are grouped together within these streams. *Cognitive-emotional resonances* support conscious percepts of feelings, as well as learning and recognition of the objects or events that cause these feelings. Cognitive-emotional resonances can synchronize with resonances that support conscious qualia and knowledge about them.

These resonances embody parametric properties of individual conscious experiences that enable effective actions to be chosen without interference from earlier processing stages. For example, surface-shroud resonances help to control looking and reaching; stream-shroud resonances help to control auditory communication, speech, and language; and cognitive-emotional resonances help to acquire valued goal objects. In autonomous adaptive systems that solve the stability-plasticity dilemma using ART dynamics, formal mechanistic homologs of such different states of resonant consciousness may be needed to choose the different kinds of actions that they control. More information will be summarized below about cognitive-emotional resonances in sections 6.5–6.7.

## 5. Building Upon Three Basic Design Themes: Homologous Circuits for Reaching and Navigating

A third design theme that is realized by the SOVEREIGN model is that advanced brains use homologous circuits to compute arm movements during reaching behaviors, and body movements during spatial navigation. In particular, both navigational movements and arm movements are controlled by circuits which share a similar mismatch learning law—called a Vector Associative Map, or VAM (Gaudiano and Grossberg, 1991, 1992; see section 5.3)—that enables learned calibration of *difference vectors* in the manner described below. This proposed homology clarifies how navigational and arm movements can be coordinated when a body navigates toward a goal object before grasping it. SOVEREIGN used difference vectors to model navigational movements. It did not, however, include a controller for arm movements that could grasp a valued object when it came within range. The text below indicates how unsupervised incremental learning in SOVEREIGN2 realizes such a capability and can, moreover, do so using a tool (see section 5.4).

### 5.1. Arm Movement Control Using Difference Vectors and Volitional GO Signals

Neural models of arm movement trajectory control, such as the Vector Integration to Endpoint, or VITE, model (Bullock and Grossberg, 1988) (Figure 8, left panel) and their refinements (e.g., Bullock et al., 1993) (Figure 8, right panel) propose how cortical arm movement control circuits compute a representation of where the arm wants to move (i.e., the *target position vector T*) and subtracts from it an outflow representation of where the arm is now (i.e., the *present position vector P*). The resulting *difference vector D* between target position *T* and present position *P* represents the direction and distance that the arm needs to move to reach its goal position. Basal ganglia (BG) volitional signals of various kinds, such as a GO signal *G*, transform the difference vector *D* into a motor trajectory that can move with variable speed by multiplying *D* with *G*, before this product is integrated by *P.* Because *P* integrates the product *DG, DG* represents the commanded outflow movement speed. Then *P* moves at a speed that increases with *G*, other things being equal. As *P* approaches *T, D* approaches zero, along with the outflow speed *DG*, so the movement terminates at the desired target position.

### 5.2. Computing Present Position for Spatial Navigation From Vestibular Signals: Place Cells

Because the arm is attached to the body, the present position of the arm can be computed using outflow, or corollary discharge, commands *P* that are derived directly from the movement commands to the arm itself (Figure 8, left panel). In contrast, when a body moves with respect to the world, no such immediately available present position command is available. The ability to compute a difference vector between a target position and the present position of the body-in order to determine the direction and distance that the body needs to navigate to acquire the target-requires more elaborate brain machinery. At the time SOVEREIGN was published, computation of such a Present Position Vector, called NET in SOVEREIGN, used an algorithm to estimate the information that vestibular signals compute *in vivo*.

SOVEREIGN breaks down spatial navigation into sequences of straight excursions in fixed directions, after which a head/body turn changes the direction before another straight excursion occurs. *In vivo*, vestibular signals provide angular velocity and linear velocity signals that can be integrated to compute these head/body angles and straight movement distances. The SOVEREIGN algorithm adds the head/body turn angles, as well as the body approach distances for each straight excursion, to compute NET. Then, as Figure 1B summarizes, NET is subtracted from the Reactive Visual TPV Storage to compute a Reactive DV, which controls the next straight movement in space. Each head/body turn resets NET to allow the next NET estimate to be computed. Using such computations, SOVEREIGN was able to learn how to navigate toward valued goals in structured environments like the maze in Figure 2.

In sufficiently advanced terrestrial animals, from rats to humans, an animal’s position in space is computed from a combination of both visual and *path integration* information. The visual information is derived from 3D perceptual representations that are completed by processes such as boundary completion and surface filling-in. The path integration information is derived from vestibular angular velocity and linear velocity signals that are activated by an animal’s navigational movements. This vestibular information is transformed by entorhinal grid cells and hippocampal place cells into representations of the animal’s present position in space (O’Keefe and Nadel, 1978; Hafting et al., 2005). The GridPlaceMap model simulated how these cells learn their spatial representations as the animal navigates realistic trajectories (e.g., Grossberg and Pilly, 2014). Key properties of the GridPlaceMap model and some of the grid cell and place cell data that it can explain are summarized in section 8.

When SOVEREIGN2 replaces the algorithmic computations of NET in Figure 1B by circuits that learn grid and place cells, it can then autonomously learn spatial NET estimates as the animat navigates novel environments that may be far more complicated than the plus maze in Figure 2. When such a self-organized NET estimate is used to compute a difference vector between the present and target positions, a volitional GO signal can move the animat toward the desired target, just as in the case of an arm movement.

### 5.3. From VITE to VAM: How a Circular Reaction Drives Mismatch Learning to Calibrate VITE

In order for VITE dynamics to work properly, its difference vectors need to be properly calibrated. In particular, when *T* and *P* represent the same position in space, *D* must equal zero. However, *T* and *P* are computed in two different networks of cells. It is too much to expect that the activities of these two networks, and the gains of the pathways that carry their signals to *D*, become perfectly matched without the benefit of some kind of learning. The Vector Associative Map, or VAM model explains how this kind of learning occurs (Gaudiano and Grossberg, 1991, 1992). In brief, the VAM model corrects this problem using a form of mismatch learning that adaptively changes the gains in the *T*-to-*D* pathways until they match those in the *P*-to-*D* pathways, so that when *T* = *P, D* = *0.*

The VAM model does this using what has been called a *circular reaction* since the pioneering work of Jean Piaget on infant development (Piaget, 1945, 1951, 1952). All infants normally go through a *babbling phase*, and it is during such a babbling phase that a circular reaction can be learned. In particular, during a visual circular reaction, babies endogenously babble, or spontaneously generate, hand/arm movements to multiple positions around their bodies. As their hands move in front of them, their eyes automatically, or reactively, look at their moving hands. While the baby’s eyes are looking at its moving hands, the baby learns an associative map from its hand positions to the corresponding eye positions, *and* from eye positions to hand positions. Learning of the map between eye and hand in both directions constitutes the “circular” reaction.

After map learning occurs, when a baby, child, or adult looks at a target position with its eyes, this eye position can use the learned associative map to generate a movement command to reach the corresponding position in space. In order for the command to be read out, a volitional GO signal from the BG-notably from the substantia nigra pars reticulata, or SNr—opens the corresponding movement gate (Prescott, 2008). Such a gate-opening signal realizes “the will to act.” Then the hand/arm can reach to the foveated position in space under volitional control. Because our bodies continue to grow for many years as we develop from babies into children, teenagers, and adults, these maps continue updating themselves throughout our lives.

In a VAM, endogenous babbling is accomplished by an Endogenous Random Generator, or ERG+, that sends random signals to *P* that cause the arm to automatically babble a movement in its workspace. This movement is thus not under volitional control. When *P* gets activated, in addition to causing the arm to move, it sends signals that input an inhibitory copy of itself to *D*.

The ERG has an opponent organization. It is the ERG ON, or ERG+, component that energizes the babbled arm moment. When ERG+ momentarily shuts off, ERG OFF, or ERG-, is disinhibited and opens a gate that lets *P* get copied at *T*, where it is stored. At this moment, both *T* and *P* represent the same position in space. If the model were correctly calibrated, the excitatory *T*-to-*D* and inhibitory *P*-to-*D* signals that input to *D* in response to the same positions at *T* and *P* would cancel, causing *D* to equal zero. If *D* is not zero under these circumstances, then the signals are not properly calibrated. The VAM model uses such non-zero *D* vectors as *mismatch teaching signals* that adaptively calibrate the *T*-to-*D* signals. As perfect calibration is achieved, *D* approaches zero, at which time mismatch learning self-terminates.

Another refinement of VITE showed how arm movements can compensate for variable loads and obstacles, and interpreted the hand/arm trajectory formation stages in terms of identified cells in motor and parietal cortex, whose temporal dynamics during reaching behaviors were quantitatively simulated (Bullock et al., 1998; Cisek et al., 1998).

### 5.4. Motor-Equivalent Reaching With Clamped Joints and Tools: The DIRECT Model

Yet another VITE model refinement, called the DIRECT model (Figure 8, right panel), builds upon VAM calibration to propose how *motor-equivalent reaching* is learned (Bullock et al., 1993). Motor-equivalent reaching explains how, during movement planning, either arm, or even the nose, could be moved to a target position, depending on which movement system receives a GO signal.

The DIRECT model also begins to learn by using a circular reaction that is energized by an ERG (Figure 8, right panel). Motor-equivalent reaching emphasizes that reaching is not just a matter of combining visual and motor information to transform a target position on the retina into a target position in body coordinates. Instead, these visual and motor signals are first combined to learn a representation of the *space* around the actor which can then be downloaded to move any of several motor effectors.

Remarkably, after the DIRECT model uses its circular reaction to learn its spatial representations and transformations, its motor-equivalence properties enable it to accurately move an arm, even when its joints are clamped, to any position in its workspace on the first try. DIRECT can also *manipulate a tool in space*. The conceptual importance of this result cannot be overemphasized: Without measuring tool length or angle with respect to the hand, the model can move the tool’s endpoint to touch the target’s position correctly under visual guidance *on its first try*, in a single reach without later corrective movements, and without additional learning. In other words, the *spatial affordance* for tool use, a critical foundation of human societies, follows from the brain’s ability to learn a circular reaction for motor-equivalent reaching in space. Adding these reaching capabilities to SOVEREIGN2 will enable it to use tools to manipulate target objects after it navigates to them.

### 5.5. Social Cognition: Joint Attention and Imitation Learning Using CRIB Circular Reactions

The DIRECT model shows how the spatial affordance for tool use could arise as a result of the circular reactions that enable reaching behaviors to develop. With DIRECT on board, a child, monkey, or robot could then volitionally reach objects with its own hand, or even using a tool like a stick. If a monkey happened to pick up a stick in this way, put it into an ant hill, and pulled it out with some ants on it, it could learn this skill to eat ants in the future whenever it wanted to do so. However, another monkey looking at this skill could not learn it from the first one without further brain machinery, because the two monkeys experience this event from two different spatial vantage points. This additional brain machinery is needed for social cognitive skills to be learned, including the learning of joint attention and imitation learning. These are competences upon which all human societies have built.

Grossberg and Vladusich (2010) develops the Circular Reactions for Imitative Behavior, or CRIB, model to explain how imitation learning utilizes *inter*-personal circular reactions that take place between teacher and learner, notably how a learner can follow a teacher’s gaze to fixate a valued goal object, and distinguishes them from the classical *intra*-personal circular reactions of Piaget that take place within a single learner, such as the one that enables reaching behaviors to be learned. After a learner can volitionally reach objects on its own, it can also learn, using an inter-personal circular reaction, to reach an object at which a teacher is looking, such as a stick with which to retrieve ants from an anthill. By building upon intra-personal circular reactions that are capable of learning motor-equivalent reaches, the CRIB model hereby clarifies how a pupil can learn from a teacher to manipulate a tool in space.

In order to achieve joint attention and imitation learning, the learner needs to be able to bridge the gap between the teacher’s coordinates and its own. In the neurobiological literature, this capability is often attributed to *mirror neurons* that fire either if an individual is carrying out an action or just watching someone else perform the same action (Rizzolatti and Craighero, 2004; Rizzolatti, 2005). This attribution does not, however, mechanistically explain how the properties of mirror neurons arise. The CRIB model proposes that the “glue” that binds these two coordinate systems, or perspectives, together is a surface-shroud resonance. How this works is modeled in Grossberg and Vladusich (2010). It is also known that a breakdown of joint attention can cause severe social difficulties in individuals with autism. How these and other breakdowns in learning cause symptoms of autism are modeled by the iSTART model (Grossberg and Seidman, 2006).

If CRIB-like social cognition capabilities are incorporated into a “classroom” of SOVEREIGN2 robots, they can then all learn sensory-motor skills from a teacher who they see from different vantage points.

### 5.6. Platform Independent Movement Control

If SOVEREIGN2 is used to control an embodied mobile robot, then an important design choice is whether to use legs or wheels with which to navigate. Difference vector (DV) control of direction and distance that is gated by a GO signal can be used in either case.

To help guide the development of a legged robot, neural network models have shown how leg movements can be performed with different gaits, such as walk or run in bipeds, and walk, trot, pace, and gallop in quadrupeds, as the GO signal size increases (Pribe et al., 1997).

An example of DV-GO control in a wheeled mobile robot was developed by Zalama et al. (1995) and Chang and Gaudiano (1998) and tested on robots such as the Khepera and Pioneer 1 mobile robots to demonstrate VAM learning of how to approach rewards and avoid obstacles in a cluttered environment, with no prior knowledge of the geometry of the robot or of the quality, number, or configuration of the robot’s sensors. Learning in one environment generalized to other environments because it is based on the robot’s egocentric frame of reference. The robot also adapted on line to miscalibrations produced by wheel slippage, changes in wheel sizes, and changes in the distance between the wheels.

In summary, both navigational movements in the world and movements of limbs with respect to the body use a difference vector computational strategy.

Sections 6–8 provide a deeper and broader conceptual and mechanistic insight into the themes that the earlier sections have introduced.

## 6. Resonant Dynamics for Perception, Cognition, Affect, and Planning

### 6.1. Invariant Object Category Learning Uses Feature-Category Resonances and Surface-Shroud Resonances

**FIGURE 7:**
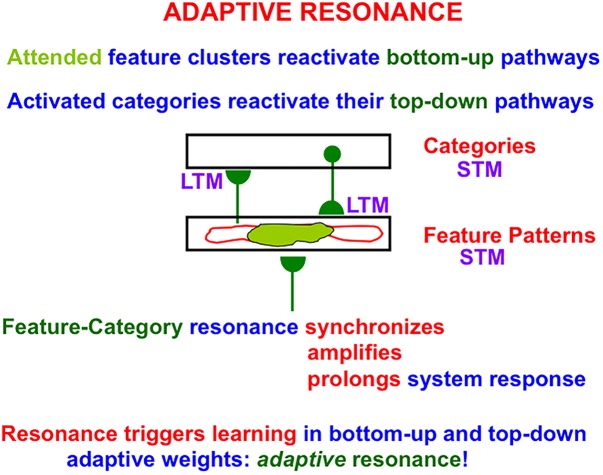
During an adaptive resonance, attended feature patterns interact with recognition categories, both stored in short-term memory (STM), via positive feedback pathways that can synchronize, amplify, and prolong the resonating cell activities. Such a resonance can trigger learning in the adaptive weights, or long-term memory (LTM) traces, within both the bottom-up adaptive filter pathways and the top-down learned expectation pathways. In the present example, the resonance is a *feature-category resonance* (see Table 1a).

Many of the enhanced capabilities of SOVEREIGN2 will use resonant processes. In particular, in order for SOVEREIGN2 to learn view-, position-, and size-invariant object categories as it scans a scene with eye, or camera, movements, two different types of resonances need to be coordinated: feature-category resonances and surface-shroud resonances. *In vivo*, view-, position-, and size-specific visual percepts in the striate and prestriate visual cortices V1, V2, and V4 are transformed into view-, position-, and size-specific object recognition categories in the posterior inferotemporal cortex (ITp) via *feature-category resonances* (Table 1a and Figure 7) within the What cortical stream.

**FIGURE 8:**
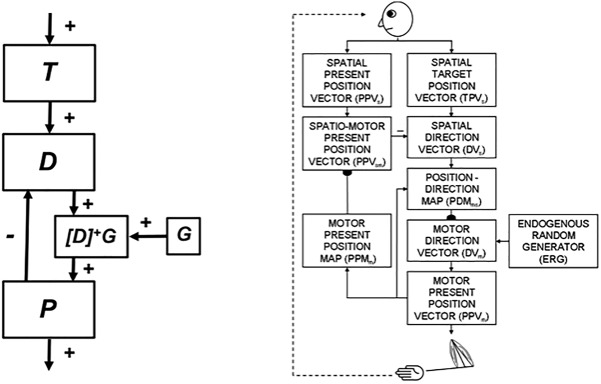
**(Left)** Vector Integration To Endpoint, or VITE, model circuit for reaching. A present position vector (*P*) is subtracted from a target position vector (*T*) to compute a difference vector (*D*) that represents the distance and direction in which the arm must move. The rectified difference vector (*[D]*), where [*D*] = max(*D*, 0), is multiplied by a volitional GO signal (*G*) before the velocity vector [*D*]*G* is integrated by *P* until *P* equals *T*, hence the model name Vector Integration to Endpoint. [Adapted with permission from Bullock and Grossberg (1988)]. **(Right)** DIRECT model circuit. This refinement of VITE processing enables the brain to carry out *motor equivalent* reaching. DIRECT can move a tool under visual guidance to its correct endpoint position on the first try, without measuring the dimensions of the tool or the angle that it makes with the hand. DIRECT hereby clarifies how a spatial affordance for tool use may have arisen from the ability of the brain to learn reaches in space during infant development. An endogenous random generator, or ERG, provides the “energy” to drive motor learning during a critical developmental period of motor babbling. The ERG activates a motor direction vector (DVm) that moves the hand/arm via the motor present position vector (PPVm). As the hand/arm moves, the eyes reactively track the position of the moving hand, and thereby compute the visually activated spatial target position vector (TPVs) and the spatial present position vector (PPVs). These vectors, which coincide during reactive tracking, are used to compute the spatial difference vector (DVs). This spatial transformation, along with the mapping from spatial directions into motor directions, gives the model its motor equivalent reaching capabilities. To compute them, the PPVs activates the spatio-motor present position vector (PPVsm), which is subtracted from the TPVs. As a result, the PPVs signal that reaches the TPVs is slightly delayed, thereby enabling the DVs computation to occur. The PPVsm stage is one of two stages in the model where spatial and motor representations are combined. The subscripts “s” and “m” denote spatial and motor, respectively. A transformation, called a circular reaction (Piaget, 1945, 1951, 1952), is learned from spatial-to-motor and motor-to-spatial representations at two adaptive pathways that are denoted by hemispherical synapses. The spatial direction vector (DVs) is hereby adaptively mapped into the motor direction vector (DVm) to transform visual Direction Into joint Rotation that gives the DIRECT model its name. [Reprinted with permission from Bullock et al. (1993)].

Within SOVEREIGN, the specific categories in ITp were learned using the unsupervised Fuzzy ART model (Figure 4A). Fuzzy ART can also be used for this purpose in SOVEREIGN2, with visual inputs now coming from 3D boundary and surface representations. Recognition learning may be supervised by replacing Fuzzy ART with Fuzzy ARTMAP (Carpenter et al., 1992) or any similar dynamical or algorithmic supervised version of ART. As will be summarized below, however, truly autonomous *invariant* object category learning that avoids the algorithmic tricks of SOVEREIGN will require more sophisticated network interactions.

**FIGURE 9:**
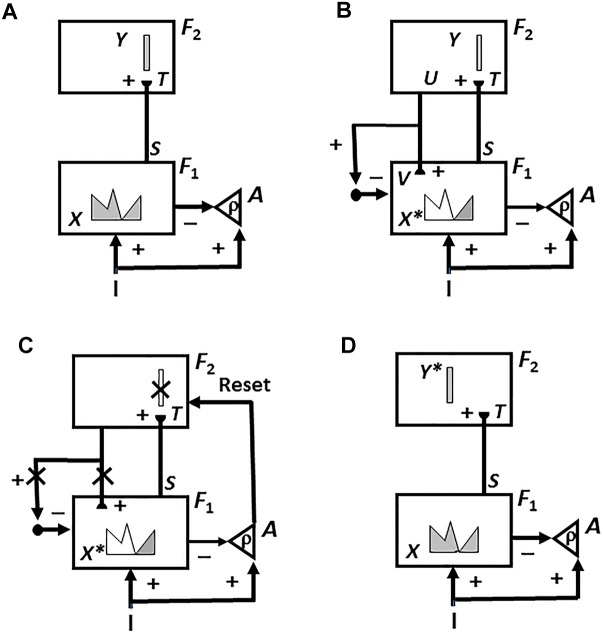
ART cycle of match-induced resonant learning and mismatch-induced reset and search. **(A)** The input pattern *I* is instated across feature detectors at level *F*_1_ as an activity pattern *X*, as it also inputs to the orienting system *A* with a gain ρ called *vigilance*. Activity pattern *X* sends inhibitory signals to *A* and a bottom-up excitatory input pattern *S* to the category level *F*_2_. Balanced excitatory inputs from *I* and inhibitory inputs from *X* keeps *A* quiet. *S* inputs are multiplied by learned adaptive weights to define the input pattern *T* to *F*_2_. Inputs *T* are contrast-enhanced and normalized within *F*_2_ by recurrent lateral inhibitory signals that obey the membrane equations of neurophysiology, also called shunting interactions. A small number of cells within *F*_2_ that receive the largest inputs are chosen by this competition. These cells represent the category Y that codes the feature pattern at *F*_1_. A winner-take-all category is shown. **(B)** Category *Y* generates top-down signals *U* that are multiplied by adaptive weights to form a prototype, or critical feature pattern, *V*. *V* represents the expectation that *Y* has learned of the feature pattern to expect at *F*_1_. If *V* mismatches *I* at *F*_1_, then a new STM activity pattern *X^∗^* (the hatched pattern), is chosen at cells where the patterns match well enough; that is, *X^∗^* is active at *I* features that are confirmed by *V*. Mismatched features (white area) are inhibited. When *X* changes to *X^∗^*, total inhibition decreases from *F*_1_ to *A*. **(C)** If inhibition decreases sufficiently, *A* triggers non-specific arousal to *F*_2_, thereby instantiating that “novel events are arousing.” Vigilance ρ determines how bad a match will be tolerated before non-specific arousal is triggered. Arousal initiates a memory search for a better-matching category in the following way: First, arousal resets *F*_2_ by inhibiting *Y.* **(D)** After *Y* is inhibited, *X* is reinstated and *Y* stays inhibited as *X* activates a different category *Y^∗^* at *F*_2_. Search continues until a better matching, or novel, category is selected. When search ends, a resonance develops that supports learning of the attended data in the adaptive weights within both the bottom-up and top-down pathways. After learning, inputs *I* can activate the globally best-matching categories directly through the adaptive filter without activating the orienting system. [Adapted with permission from Carpenter and Grossberg (1993)].

Despite its simplicity, Fuzzy ART is an algorithmic realization of dynamical properties of ART that embody both a feature-category resonance (Figure 6) and a classical example of complementary computing. Complementary computing enables feature-category resonances to continuously learn to recognize novel objects using interactions between an *attentional system* in which category learning occurs, and an *orienting system* that drives memory searches and hypothesis testing for novel categories in response to large enough mismatches between bottom-up and top-down input patterns (Figure 9) (Grossberg, 1976b, 1980, 2017).

### 6.2. Complementary Computing: ART Hypothesis Testing and Learning of Predictive Categories

The need for an orienting system can be seen by answering the question: If learning can occur only if there is a sufficiently good match between bottom-up input patterns and top-down expectations, then how is anything truly novel ever learned? Here is where complementary properties of attentional matching and orienting search are crucial: A sufficiently bad mismatch between an active top-down expectation and a bottom-up input, say because the input is unfamiliar, can drive a memory search and hypothesis testing. Such a mismatch within the attentional system activates the complementary orienting system, which is sensitive to unexpected and unfamiliar events. The ART attentional system includes the inferotemporal and prefrontal cortices, whereas the orienting system includes the non-specific thalamus and hippocampal system. See Carpenter and Grossberg (1993) and Grossberg and Versace (2008) for supportive neurobiological data.

The fact that ART learns only if a sufficiently good match occurs also imposes constraints upon how top-down adaptive weights are initially chosen to enable category learning to get started: In any ART system, the top-down adaptive weights that represent learned expectations need to be broadly distributed and large before learning occurs, so that they can match whatever input pattern first initiates learning of a new category. Indeed, when a new category is first activated, it is not known at the category level what pattern of features caused the category to be activated. *Whatever* feature pattern was active needs to be matched by the top-down expectation on the first learning trial, so that resonance and weight learning can begin. Hence the need for the initial values of top-down weights to be broadly distributed and sufficiently large to match *any* feature pattern.

Given that top-down weights are initially broadly distributed, the learning of top-down expectations is a process of *pruning* weights on subsequent learning trials, and uses mismatch-based reset events to discover categories capable of representing the environment. The large initial adaptive weights in top-down expectations helps to explain otherwise mysterious neurobiological data, such as why there is an Inverted-U through time in the power of beta oscillations when an animal first navigates a new maze (Berke et al., 2008; Grossberg, 2009a).

### 6.3. Complementary PN and N200 Event Related Potentials During Attention and Memory Search

In contrast to the *top-down, adaptive, specific*, and *match* properties that occur during an attentive match, an orienting system mismatch is *bottom-up, non-adaptive, non-specific*, and *mismatch*: A mismatch occurs when *bottom-up* activation of the orienting system cannot be adequately inhibited by the bottom-up inhibition from the matched pattern (Figure 9B). The signals to and from the orienting system are *non-adaptive*, or not subject to learning. Mismatch-activated output from the orienting system *non-specifically* arouses all the category cells because the orienting system cannot determine which categories read out the expectation that led to mismatch (Figure 9C). *Any* category may be responsible, and may thus need to be reset by arousal (Figure 9D). Finally, the orienting system is activated by a sufficiently big *mismatch*.

These are properties of the N200 event-related potential, or ERP (Näätänen et al., 1982; Sams et al., 1985). More generally, during an ART memory search, sequences of the predicted mismatch, arousal, and reset events occur that exhibit properties of the sequentially occurring P120, N200, and P300 ERPs, respectively (Banquet and Grossberg, 1987).

In summary, four sets of properties of the attentional system are complementary to those of the orienting system (top-down vs. bottom-up, adaptive vs. non-adaptive, specific vs. non-specific, match vs. mismatch), with the PN and N200 ERPs illustrating these complementary properties. The orienting system can detect that an error has occurred, but does know what category prediction caused it. The attentional system knows what categories are active, but not if these categories adequately represent current inputs. By interacting, these systems can determine what the error is and discover and learn a new category to correct it. Complementary computing hereby accomplishes incremental learning and autonomous error correction of a large non-stationary database, without incurring the risk of catastrophic forgetting.

### 6.4. Autonomous Solution of the Invariant Pattern Recognition Problem During Active Vision

In our brains, as ITp categories are learned using feature-category resonances, they create the substrate for learning view-, position-, and size-invariant object recognition categories within the ventral What cortical processing stream, notably in the anterior inferotemporal cortex, or ITa. The 3D ARTSCAN Search model has been incrementally developed to explain in detail how our brains learn to solve the invariant pattern recognition problem during active vision, a problem that is just as important for human survival as it is for designing machine learning algorithms that can autonomously learn in the real world (Fazl et al., 2009; Cao et al., 2011; Grossberg et al., 2011, 2014; Foley et al., 2012; Chang et al., 2014; Grossberg, 2017). When it is implemented in SOVEREIGN2, the 3D ARTSCAN Search architecture can be used to provide previously unavailable machine learning, recognition, and prediction abilities in autonomous adaptive mobile systems, notably self-training robots.

To carry out effective invariant category learning, the model needed to solve a basic View-to-Object Binding Problem, which concerns how our brains automatically know, without external supervision or prior learning, which views of a novel scene belong to the same object-and thus can be associated with the same invariant category-and which do not-so should not be associated. As a result, the model can learn invariant object categories in response to arbitrary combinations of unsupervised and supervised learning trials as the eyes freely scan a complex scene.

**FIGURE 10:**
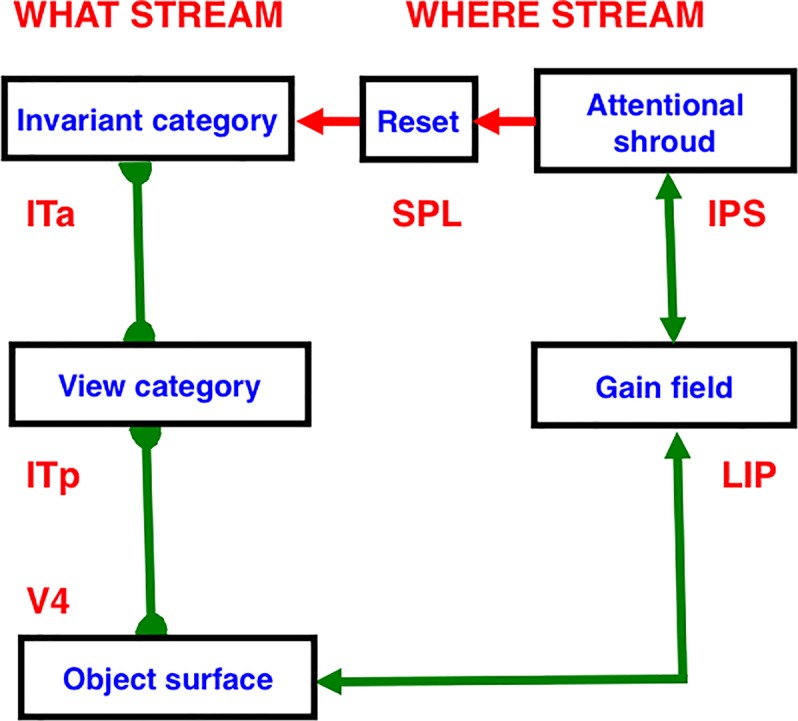
Learning of view-invariant categories in the What cortical stream is modulated by surface-shroud resonances in the Where cortical stream. The surface-shroud resonance prevents the invariant category from being reset as multiple view-specific categories are learned and associated with it as the eyes scan an attended object surface. See the text for details.

As ITp categories are learned using feature-category resonances (Figure 7), they are associated with cells in the anterior inferotemporal (ITa) cortex that learn to become view-, position-, and size-invariant object recognition categories. Figure 10 illustrates how the View-to-Object Binding Problem is solved during invariant object category learning in ITa within the What cortical stream, with the help of modulation by the PPC in the Where cortical stream, including the inferior parietal sulcus (IPS), the lateral intraparietal area (LIP), and the medial superior parietal lobule (SPL). Surface-shroud resonances that are triggered between V4 and IPS play a critical role in modulating this invariant category learning modulation process, while they also support conscious visibility of the attended object surface.

An active surface-shroud resonance embodies the brain state that maintains spatial attention upon the object that is being learned about. While the object is attended, its shroud also inhibits category reset cells in SPL (Figure 10). While the surface-shroud resonance maintains attention on an object surface, it also regulates eye movements that successively foveate the most salient features on the attended surface (not shown in Figure 10), but not other objects in the scene, thereby solving the View-to-Object Binding Problem. Each foveation can lead to the learning of a different specific ITp category. The first such ITp category to be learned chooses cells in ITa with which it will be associated via typical ART dynamics (Figure 10). As successive ITp categories are learned, they can all be associated with the same ITa cells because they cannot be inhibited by SPL. These ITa calls hereby learn to become an invariant object category by being associated with multiple specific ITp categories.

When spatial attention shifts from the object, its shroud collapses, thereby disinhibiting the reset cells in SPL. A transient burst of inhibition from these SPL cells resets the active invariant object category in ITa (Chiu and Yantis, 2009; Fazl et al., 2009). As the invariant object category collapses and the eyes attend another object’s surface, new specific ITp and invariant ITa object categories can be learned to represent other objects in a scene. The cycle can then repeat itself. The model can hereby autonomously learn invariant object categories in response to arbitrary combinations of unsupervised and supervised learning trials as its eyes or cameras are directed to scan a complex scene.

After invariant categories are learned, the system can also solve the Where’s Waldo Problem; that is, it can search a scene for a desired goal object within it. Such a search requires What-to-Where stream interactions.

### 6.5. Conditioned Reinforcer and Motivational Learning Use Cognitive-Emotional Resonance

**FIGURE 11:**
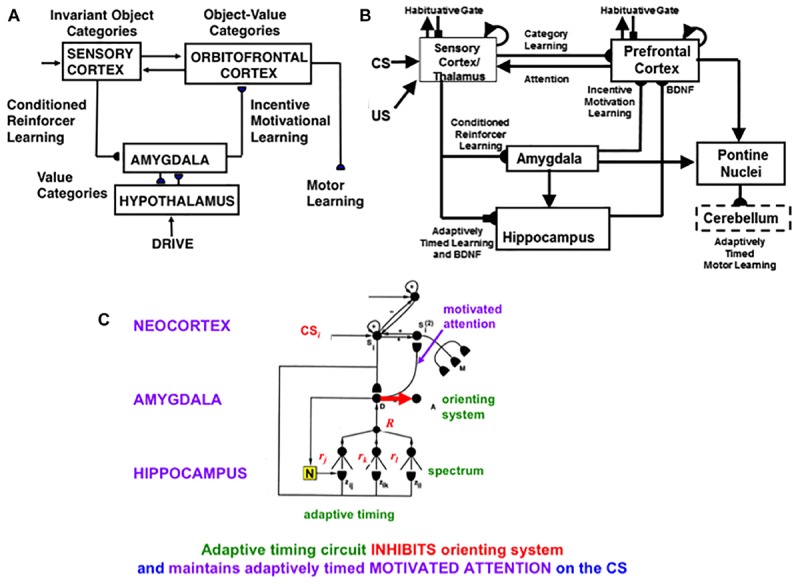
**(A)** Cognitive-Emotional-Motor (CogEM) model macrocircuit. CogEM models how invariant object categories in sensory cortex can activate value categories, also called drive representations, in the amygdala and hypothalamus, and object-value categories in the orbitofrontal cortex. Converging activation from an object category and its value category can fire the corresponding object-value category. An invariant object category can activate an object-value category by itself if prior conditioned reinforcer learning and incentive motivational learning strengthen the pathways that pass through the value category. An active object-value category sends positive feedback to sensory cortex that enhances the activity of its invariant object category. This motivationally enhanced object representation can then better compete with other object representations via a recurrent competitive network (not shown) and draw attention to itself. Maintaining feedback between object, value, and object-value categories via a cognitive-emotional resonance can induce a conscious percept of having a particular feeling about the attended object, as well as knowing what it is. The active object-value category can also generate output signals to activate cognitive expectations and actions through other brain circuits. [Adapted from Grossberg (1971) and subsequent CogEM articles]. **(B)** Macrocircuit of the neurotrophic Spectrally Timed Adaptive Resonance Theory, or nSTART, model. The sensory cortex sends signals to the prefrontal cortex, notably the inferotemporal cortex, as in **(A)**. In addition to the connections between these regions and the amygdala, nSTART also includes adaptively timed inputs from the sensory cortex to the hippocampus, which then inputs to prefrontal cortex. A similar circuit (not shown) connects thalamus to sensory cortex, amygdala, and hippocampus. nSTART also includes adaptive connections from thalamus to sensory cortex, and from sensory cortex to orbitofrontal cortex, that support object category learning. An adaptively timed cortico-hippocampal resonance can maintain the cognitive-emotional resonance that passes through amygdala, thereby supporting conscious feelings and awareness of the objects that cause them. The pontine nuclei serve as a final common pathway for reading-out conditioned responses. Cerebellar dynamics are not simulated in nSTART. Key: arrowhead = excitatory synapse; hemidisc = adaptive weight; square = habituative transmitter gate; square followed by a hemidisc = habituative transmitter gate followed by an adaptive weight. See the text for details. [Reprinted with permission from Franklin and Grossberg (2017)]. **(C)** In the START model, conditioning, attention, and timing are integrated. Adaptively timed hippocampal signals *R* maintain motivated attention via a cortico-hippocampal-cortical feedback pathway, at the same time that they inhibit activation of orienting system circuits *A* via an amygdala drive representation *D*. The orienting system *A* is also assumed to occur in the hippocampus. The adaptively timed signal is learned at a spectrum of cells whose activities respond at different rates *r_j_* and are gated by different adaptive weights *z_ij_.* A transient Now Print learning signal *N* drives learned changes in these adaptive weights. In the nSTART model in **(B)**, the hippocampal feedback circuit operate in parallel to the amygdala, rather than through it. See the text for details. [Adapted with permission from Grossberg and Merrill (1992)].

Invariant object categories in ITa (sensory cortex in Figure 11A) learn to activate value categories via conditioned reinforcer pathways, whereas value categories learn to activate object-value categories in the orbitofrontal cortex (OFC) via incentive motivational pathways. Both kinds of learning occur during a *cognitive-emotional resonance* that is triggered when a conditioned stimulus, such as a buzzer sound, activates its invariant object category while an unconditioned stimulus, or primary reward such as presentation of food to a hungry animal, activates its value category.

A cognitive-emotional resonance begins when object-value categories fire in response to converging inputs from sensory cortex and a value category. Then top-down feedback from the object-value category to its invariant object category closes a feedback loop between sensory cortex, amygdala (AMYG), and OFC that supports the cognitive-emotional resonance. This kind of resonance focuses motivated attention upon valued objects, while triggering context-appropriate actions toward them.

The model in Figure 11A that accomplishes conditioned reinforcer learning, incentive motivational learning, and release of motor actions toward valued goal objects is called the Cognitive-Emotional-Motor, or CogEM, model. CogEM has been getting incrementally developed since it was introduced in 1971 (e.g., Grossberg, 1971, 1982, 1984b; Grossberg and Gutowski, 1987; Dranias et al., 2008). The drive representations of the CogEM model include opponent processing channels called *gated dipoles* (Grossberg, 1972a,b, 1984b) that organize affective processing into opponent channels such as fear vs. relief, and hunger vs. frustration, which help to regulate behaviors like approach vs. avoidance, and exploration vs. consummation (cf. exploration vs. exploitation). Each gated dipole controls the balance between one pair of opponent affective representations. Variations of the gated dipole design occur in multiple brain processes, including the representation of opponent colors such as red vs. green, opponent directions such as up vs. down, and opponent muscles such as agonists vs. antagonists. Gated dipoles are thus a general design that helps to *reset* brain dynamics in response to sudden changes in environmental contingencies, and to restore brain dynamics to an unbiased state.

### 6.6. Antagonistic Rebounds Enable Opponent Extinction and Learning From Disconfirmations

Gated dipole reset takes the form of an *antagonistic rebound* during which activation in its ON channel is replaced by a transient activation, or rebound, in its OFF channel. An antagonistic rebound can be triggered in response to a sudden decrease in the phasic input that was activating the ON channel, or to an unexpected event that causes a sudden increase in the arousal that activates both the ON and OFF channels (Grossberg, 1984b; Grossberg and Schmajuk, 1987). In this way, changing environmental contingencies, including the disconfirmation of expected events, can have reinforcing properties that can modulate which learned plans will be chosen to triggered goal-oriented actions in a particular environmental context.

When adaptive weights learn from both ON channel activations and OFF channel rebounds in response to disconfirmations of previous learning, then approximately equal learned inputs to both the ON and OFF channels can occur and lead to competitive suppression of output signals. The emotional and motivational support for such behaviors is then eliminated; the behavior has been *extinguished*. Recurrent gated dipoles called READ circuits, for Recurrent Associative Dipole, enable opponent learning and extinction to go on throughout life, without ever saturating the learned weights, no matter how many learning and extinction trials they may experience (Grossberg and Schmajuk, 1987).

**FIGURE 12:**
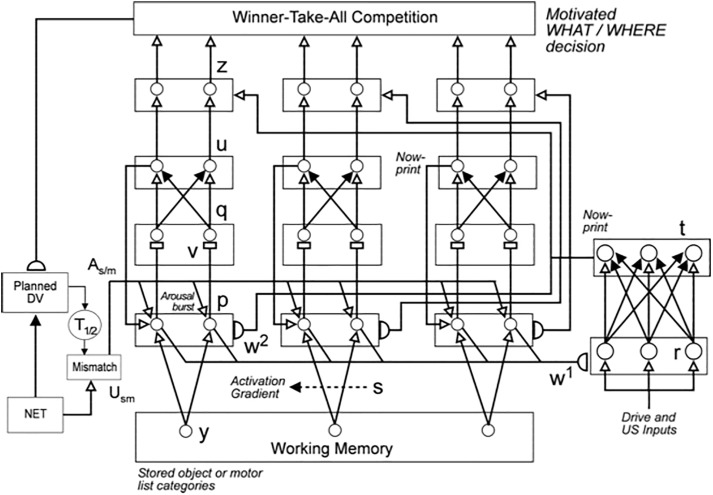
The Gated Multipole network includes multiple gated dipoles that regulate reinforcement learning and incentive motivational learning to help chose those object and spatial list chunks that control actions which predict the most valued outcomes in the current environment. See the text for details. [Reprinted with permission from Gnadt and Grossberg (2008)].

SOVEREIGN models an array of gated dipoles, called gated multipoles (Figure 4, 12), in which multiple opponent affective states compete with each other to decide which one of them has the momentarily best combination of sensory and motivational inputs to control behavioral choices as environmental conditions change. Gated multiples within CogEM circuits will also occur in SOVEREIGN2.

### 6.7. Adaptively Timed Cortico-Hippocampal Resonances Support Learning Across Temporal Gaps

Learning often requires that learned associations form between sensory cues and reinforcers that are separated in time, with the sensory cues shutting off hundreds of milliseconds or even seconds before the reinforcer turns on. The CogEM model cannot learn in such situations because the AMYG cannot bridge temporal gaps of such a long duration. *In vivo*, the hippocampus (HIPPO) enables conditioning to bridge temporal gaps using a type of adaptively timed learning (Figure 11B) that is called *spectral timing* (Grossberg and Schmajuk, 1989; Grossberg and Merrill, 1992, 1996). Spectrally timed learning can bridge time intervals of hundreds of milliseconds between the offset of a conditioned stimulus (CS) and the onset of a rewarding unconditioned stimulus (US), as occurs during reinforcement learning paradigms like trace conditioning and delayed-non-match to sample. It does so using populations of cells that each respond at different times (the “spectrum”), but for much shorter time intervals than the population response as a whole can span.

How do neurons, which typically fire on a millisecond time scale, span hundreds of milliseconds? Fiala et al. (1996) developed a detailed spectral timing model of cerebellar adaptive timing that links biochemistry, neurophysiology, neuroanatomy, and behavior, and predicts how the metabotropic glutamate (mGluR) receptor system may create a spectrum of delays during cerebellar adaptively timed learning. mGluRs are a form of glutamate receptor that is different from the ionotropic glutamate receptors that support widespread excitatory signaling throughout the brain. Unlike ionotropic glutamate receptors, which directly activate ion channels, mGluR receptors activate biochemical cascades. Spectral timing properties are predicted to be an example of such a biochemical cascade, with intracellular calcium regulating the different response rates of the cells within such a spectrum. This prediction has been supported by several subsequent experiments (e.g., Finch and Augustine, 1998; Takechi et al., 1998; Ichise et al., 2000; Miyata et al., 2000).

In addition to the mGluR spectral timing circuits that have modeled adaptively timed actions using the cerebellum (Fiala et al., 1996), similar mGluR circuits have modeled maintenance of adaptively timed incentive motivation that supports such actions using the HIPPO (Grossberg and Schmajuk, 1989; Grossberg and Merrill, 1992, 1996), and adaptively timed reinforcement learning in response to unexpected rewards and punishments using the BG (Brown et al., 1999, 2004). Indeed, variants of spectral timing seem to be an ancient evolutionary discovery that includes non-neural systems. Simpler versions of such calcium-modulated spectra also occur in non-neural tissues such as HeLa cancer cells (Bootman and Berridge, 1996), the *puffs* in Xenopus oocytes (Yao et al., 1995), and the *sparks* in cardiac myocytes (Cannell et al., 1995; López-López et al., 1995).

In particular, the Spectrally Timed Adaptive Resonance Theory, or START, model has explained and simulated how spectrally timed learning may occur in dentate-hippocampal circuits (Figure 11C) (Grossberg and Schmajuk, 1989; Grossberg and Merrill, 1992, 1996). Data about both normal and abnormal learned timing have been explained by this model, including explanations of timing failures in individuals with autism and Fragile X syndrome (Grossberg and Seidman, 2006; Grossberg and Kishnan, 2018).

The neurotrophic START, or nSTART, model (Figure 11B) developed hippocampal spectral timing properties a different direction by proposing how spectral timing supports memory consolidation of previously learned associations using a combination of endogenous hippocampal bursting and modulation by brain-derived neurotrophic factor, or BDNF, during the consolidation period, which often occurs during periods of sleep (Franklin and Grossberg, 2017). If the HIPPO is ablated shortly after learning, then memory consolidation cannot take place, and medial temporal amnesia can be caused. More generally, the nSTART model explains and simulates why lesions of thalamus, AMYG, HIPPO, and OFC have different effects on memory consolidation, depending on the phase of learning when they occur.

Both START and nSTART explain how a *cortico-hippocampal resonance* sustains cognitive-emotional resonances using its adaptively timed learning long enough for brains to become conscious of feelings and the events that caused them. The pART model circuit in Figure 5 includes spectrally timed interactions between anterior inferotemporal cortex (ITa), HIPPO, and OFC, which then closes the adaptively timed feedback loop with ITa.

Adaptively timed behaviors are essential for success in an autonomous adaptive mobile system, including learning to properly time goal-oriented actions and to maintain motivated attention upon desired goal objects long enough to do so. A model HIPPO and cerebellum can be joined to the CogEM multipole model to enable SOVEREIGN2 to learn and control both of these kinds of adaptively timed behaviors.

### 6.8. Expected vs. Unexpected Disconfirmations Regulate Consummation vs. Exploration

Combining ART and START circuits into a larger architecture enables a brain to adaptively cope with situations wherein cues that have led to expected consequences in the past no longer do so. In particular, it enables humans to wait for delayed rewards, yet also prevents perseveration of behaviors to acquire a goal that is no longer forthcoming, with possibly disastrous consequences, such as starvation if food is no longer available. This competence is achieved by distinguishing *expected disconfirmations*-also called *expected non-occurrences-*of reward from *unexpected disconfirmations*-or *unexpected non-occurrences*-of reward.

In particular, why do not animals treat expected non-occurrences of reward as predictive failures? Why do they not always become frustrated by the immediate non-occurrence of a reliable reward that is typically delayed in time, and trigger exploratory behavior to find it elsewhere, leading to relentless exploration for immediate gratification? And if animals do wait, but the reward does not appear at the expected time, how does the animal adaptively respond to the unexpected non-occurrence of the reward-that is, to the occurrence of *nothing*? In normal animals, expected disconfirmations do not prevent acquisition of a delayed reward, even though unexpected disconfirmations can trigger reset of working memory, attention shifts, frustrative rebounds that can extinguish unsuccessful gated dipole associations, and the release of exploratory behaviors to discover better sources of the desired goal object.

In either case, if the reward happens to occur earlier than expected, the animal could still perceive it via a cognitive-emotional resonance and release a consummatory response. Thus, the registration of ART-like sensory matches is not inhibited during either expected or unexpected non-occurrences (Figure 9). However, during an expected disconfirmation, the *effects of mismatches* upon activation the ART orienting system, which cause a reduction of ART inhibition there (Figure 9B),are compensated by the addition of adaptively timed input from the HIPPO (Figure 11C). Activation of the orienting system is hereby prevented during an expected disconfirmation, and with it reset of working memory, attention shifts, frustrative rebounds, and the release of exploratory behaviors. In contrast, during an unexpected non-occurrence, the orienting system is disinhibited by the ART mismatch because the spectral timing circuit is not active then, so reset of working memory, attention shifts, frustrative rebounds, and the release of exploratory behaviors can occur with which to correct the predictive error.

A spectral timing response begins immediately after its triggering stimulus, and builds throughout the interstimulus interval, or ISI, between the CS and US (Grossberg and Schmajuk, 1989; Grossberg and Merrill, 1992, 1996). It can thus maintain inhibition of the orienting system until the expected time of occurrence of the rewarding stimulus (Figure 11C). Adaptively timed excitation can also maintain motivated attention upon the correct orbitofrontal representation throughout this time interval (Figure 11C). By peaking at the expected time of the reward, motivated attention can most probably elicit a learned response when the reward is expected.

### 6.9. Working Memories and Learning of List Chunk Plans Using Item-List Resonances

During cognitive and cognitive-emotional learning and action cycles, as an animal or animat navigates through its environment, sequences of object categories may be temporarily stored in an *object* working memory (Figure 4A) that occurs in human and other primate brains in the ventrolateral prefrontal cortex (VLPFC), at the same time that sequences of the positions/directions where they are found in a scene are temporarily stored in a *spatial* working memory (Figure 4B) in the dorsolateral prefrontal cortex (DLPFC; see Figure 5).

**FIGURE 13:**
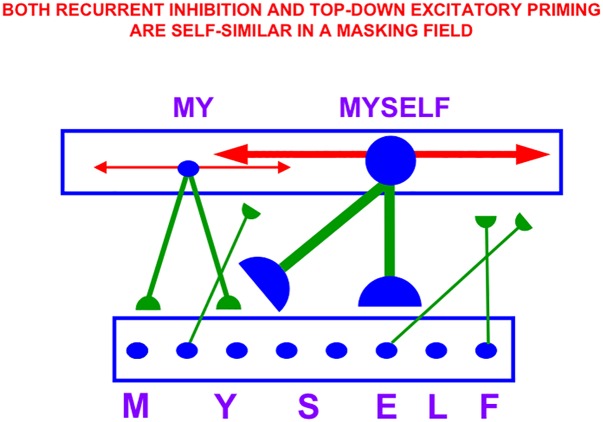
An Item-Order-Rank working memory (lower level) for the short-term sequential storage of item chunks (e.g., M, Y, S, E, L, F) can activate a multiple-scale Masking Field list chunking network (upper level) through a bottom-up adaptive filter. The larger cell sizes and interaction strengths of the list chunks that categorize longer lists (e.g., MYSELF vs. MY) enable the Masking Field to choose the list chunk that currently receives the largest total input, and thus best predicts the sequence that is currently stored in the Item-Order-Rank working memory. The chosen list chunk can then read-out the most likely prediction of what will happen next in that temporal context. Green connections are excitatory. Red connections are inhibitory. Arrowheads at the ends of Masking Field inhibitory recurrent pathways denote connections that undergo no learning. Hemidiscs denote connections that can undergo learning, both in the bottom-up filter connections and the top-down expectation connections. Recurrent on-center off-surround connections in the Item-and-Order working memory are not shown, for simplicity. Recurrent self-excitatory connections in the Masking Field are also not shown, again for simplicity. [Reprinted with permission from Grossberg (2018)].

As they are stored in working memory, object category sequences trigger learning of object plans, or *object list chunks*, while stored position/direction sequences trigger learning of spatial plans, or *spatial list chunks*, that selectively respond to the particular sequences that are stored in their working memory. A network that can learn list chunks of variable length is called a Masking Field (Figure 4) (Cohen and Grossberg, 1986, 1987; Grossberg and Kazerounian, 2011; Kazerounian and Grossberg, 2014). As illustrated in Figure 13, a Masking Field contains cells of variable size in which larger cells respond selectively to longer working memory lists. Masking Fields can learn these properties using simple laws of activity-dependent cell growth during their development, which leads to a multiple-scale network of *self-similar* cells whose cell body sizes and connection strengths covary (Cohen and Grossberg, 1987; Kazerounian and Grossberg, 2014).

The learning of list chunks by a Masking Field in SOVEREIGN used only bottom-up adaptive filter pathways (Figure 4). *In vivo*, list chunk learning is dynamically stabilized by *item-list resonances* in the corresponding parts of the PFC (Table 1a). Figure 13 illustrates the fact that the top-down learned expectation pathways that interact with bottom-up adaptive filter pathways to trigger and sustain an item-list resonance can also regulate choice of the most predictive list chunk in each environment and prime the sequences of working memory items that support that choice. Such item-list resonances in SOVEREIGN2 can greatly increase the stability of this kind of learning under multiple kinds of perturbations.

### 6.10. Masking Fields Learn List Chunks From Resonating Item-Order-Rank Working Memories

These particular working memories and list chunking networks are used because they embody fundamental design principles that are needed for autonomous adaptive storage and learning of event sequences. In particular, feedback interactions between both types of circuits solve a Temporal Chunking Problem, which concerns how a new word, motor skill, or navigational route gets learned when it is composed of familiar subsequences, without undermining previous learning of the subsequences. In the case of language, for example, suppose that the new word is composed of syllables that are themselves already familiar words. The problem is: Why is not the brain forced to process the new word as a sequence of smaller familiar words? How does a not-yet-established word representation overcome the salience of already well-learned phoneme, syllable, or word representations to enable learning of the novel word to occur? How does this occur, moreover, under unsupervised learning conditions?

For example, suppose that the words MY, ELF, and SELF have already been learned, and have their own list chunks. When the novel word MYSELF is presented for the first time, all of its familiar subwords also get presented as part of this longer sequence. What mechanisms prevent the familiarity of MY, ELF, and SELF, which are trying to activate their own list chunks, from forcing the novel longer list MYSELF from being processed as a sequence of these smaller familiar chunks, rather than eventually as a newly learned unitized whole? If this did happen, then longer words could never be learned. Nor could longer navigational routes that include familiar subroutes, or more complex motor skills that include familiar gestures. Our brains would experience a *reductio ad absurdum.* It is because the multiple scales of a Masking Field are self-similar that the larger scale that is activated by MYSELF can inhibit the smaller scales that are activated by MY, ELF, and SELF, even before the list chunk for MYSELF is tuned by category learning. The multiple self-similar spatial scales of Masking Fields hereby enable them to learn how to categorize lists of variable lengths.

Even if a novel longer list like MYSELF could overcome competition from its familiar subwords, what would prevent its new learning from forcing catastrophic forgetting of the list chunks of its familiar subwords? A solution of this problem is said to obey the LTM Invariance Principle. Item-Order-Rank working memories solve the LTM Invariance Principle (Grossberg, 1978, 2017; Bradski et al., 1992, 1994; Grossberg and Myers, 2000; Grossberg and Pearson, 2008; Grossberg and Kazerounian, 2011; Silver et al., 2011; Kazerounian and Grossberg, 2014). They store the temporal *order* of sequences of events occurring in time into an evolving spatial gradient of activities over content-addressable *item* representations that can represent items that are repeated multiple times; that is, have different *ranks* (e.g., ABACAD). Thus, Item-Order-Rank working memories can store sequences of events with repeats while satisfying the LTM Invariance Principle. They do so by preserving the *relative* activities of stored items as new items in a sequence are stored, even while the total activity of all stored items can change greatly through time.

Because all working memories need to satisfy the LTM Invariance Principle, *all* working memories, whether linguistic, motor, or spatial, were predicted to be realized by a similar kind of circuit. This circuit was shown to be a specialized version of a type of circuit that is ubiquitous in the brain; namely, a recurrent shunting on-center off-surround network, thereby clarifying how such a seemingly sophisticated design as a working memory could be discovered during evolution. Masking Fields are also recurrent shunting on-center off-surround networks, and thus are also working memories, albeit working memories that also represent list chunks.

Feedback interactions between an Item-Order-Rank working memory and a Masking Field solve the Temporal Chunking Problem, and can do so under unsupervised learning conditions. These feedback interactions trigger an *item-list resonance* that dynamically stabilizes the bottom-up list chunk learning and the learning of the top-down expectations that enable list chunks to activate sequences of events in working memory for skilled performance. Item-list resonances hereby illustrate how ART dynamics solve the stability-plasticity dilemma in the temporal domain, and include predictions about the oscillatory dynamics, including gamma and beta oscillations, that occur during these resonances in primate brains.

All of the predicted properties of Item-Order-Rank working memories have been supported by subsequent psychological data (e.g., Jones et al., 1995; Page and Norris, 1998; Farrell and Lewandowsky, 2004; Agam et al., 2005, 2007) and neurobiological data (e.g., Averbeck et al., 2002, 2003a,b; Bastos et al., 2018; Lundqvist et al., 2018).

In SOVEREIGN2, with item-list feedback signals implemented, each learned list chunk, or plan, can be selectively activated by motivationally salient sequences of previously experienced objects and positions/directions, and can then read out context-sensitive predictions of the objects and positions/directions that should be acquired next, thereby generalizing the SOVEREIGN interactions in Figure 4. This learning and performance cycle can continue through time in an unsupervised way using only the world itself as a teacher, but may also be supervised by a human teacher at arbitrary times. As noted in sections 6.5 and 6.6, CogEM includes supervision by rewards, punishments, and unexpected outcomes to drive its reinforcement learning.

### 6.11. Entorhinal-Hippocampal Resonances That Support Spatial Navigation Are Not Conscious

Yet another kind of resonance may be incorporated into SOVEREIGN2. This is the *entorhinal-hippocampal resonance* that supports learning and stable memory of entorhinal grid cells and hippocampal place cells during spatial navigation that were mentioned in section 5.2. This kind of resonance will be discussed in section 8. It illustrates the claim that, although “all conscious states are resonant states,” the converse statement is not true. In order for a resonant state to become conscious, it is necessary for it to include either representations of external sensory cues, such as visual or auditory cues, or internal sensory cues, such as emotional cues.

## 7. Prefrontal Coordination of Working Memory, Planning, and Cognitive-Emotional Dynamics

The kind of adaptive mobile intelligence that is exhibited by humans and other primates required a major expansion of the PFC to enable its working memory and planning networks to flexibly interact with multiple other brain systems, notably cognitive-emotional systems. The predictive ART, or pART, model (Figure 5) (Grossberg, 2018) has clarified how these properties arise through interactions of orbitofrontal cortex (OFC), VLPFC, and DLPFC with the inferotemporal cortex (ITp and ITa), perirhinal cortex (PRC), parahippocampal cortex (PHC), ventral bank of the principal sulcus (VPS), ventral prearcuate gyrus (VPA), frontal eye fields (FEF), hippocampus (HIPPO), amygdala (AMYG), basal ganglia (BG), hypothalamus (LH), PPC, lateral intraparietal cortex (LIP), and visual cortical areas V1, V2, V3A, V4, MT, and MST.

pART model explanations more fully embody and extend many of the processes that were included in SOVEREIGN, including how the value of visual objects and events is computed, which objects and events cause desired consequences and which may be ignored as predictively irrelevant, and how to plan and act to realize these consequences. To achieve this properties, pART includes reinforcement learning and incentive motivational learning; object and spatial working memory dynamics; and category learning, including the learning of object categories, value categories, object-value categories, and sequence categories, or list chunks. pART also explains properties that go beyond SOVEREIGN and other neural models, such as how to selectively filter expected vs. unexpected events to determine which events get stored in working memory, and how such filtering controls movements toward, and conscious perception of, expected events.

Incorporating this level of sophistication in SOVEREIGN2 will require a coordinated research program. Here primarily the new competences will be reviewed of how events can be selectively filtered before being stored in working memory, and how that ability alters the understanding of how a top-down cognitive prime from the PFC can bias object attention in the What cortical stream to anticipate expected objects and events, while it also focuses spatial attention in the Where cortical stream to trigger actions that acquire currently valued objects (Fuster, 1973; Baldauf and Desimone, 2014; Bichot et al., 2015).

### 7.1. Minimal Anatomy for Foveating Valued Objects in a Scene: Where’s Waldo?

As explained in greater detail in the pART model (Grossberg, 2018), after Where-to-What stream interactions help to learn invariant object categories, What-to-Where stream interactions regulate how to foveate valued target objects in a scene. Previous models like ARTSCAN Search and ARTSCENE Search proposed a *minimal anatomy* that could carry out this function, while also simulating challenging reaction time (RT) data about visual search for target objects (Huang and Grossberg, 2010; Chang et al., 2014). Such a minimal anatomy models how an invariant object representation in the What stream can activate a positional representation in the Where stream that can be used to foveate a valued target object in a scene. However, it did not try to solve the problem of how the brain can selectively filter desired targets from a stream that also contains distractors, so that it only attends, stores, and foveates matched targets. This additional computational property is explained by the pART model (Figure 5). However, given the ability of the minimal anatomy to quantitatively simulate challenging RT data in many visual search experiments, it may have evolved before the prefrontal mechanisms of selective working memory storage did, and may operate in parallel with them. It may be worth testing if these simpler circuits are still functional when prefrontal mechanisms are lesioned.

In the minimal anatomy of ARTSCENE Search, winning VLPFC activities send a top-down attentional prime to ITa using a circuit that obeys the ART Matching Rule. In order to transform the primed ITa cells into firing cells, an additional input must converge on ITa. This kind of signal is regulated by the BG (cf. BG in Figure 5). A volitional gate-opening signal from the BG-notably from the substantia nigra pars recitulata, or SNr-lets the primed ITa cells fire. The activated ITa cells then prime the positionally sensitive categories in ITp with which they were associated when ITa was being learned using resonant bottom-up and top-down interactions (Figure 5). If one of the primed ITp categories also receives a bottom-up input from an object at its position, then it can fire and activate positional representations in eye movement control regions like LIP and FEF. These positional representations can then move the eyes to the position in space that they represent.

### 7.2. Cortical What Working Memory Filtering and Activation of Where Target Positions

Multiple experiments show that selective working memory storage in the PFC does occur. The pART model offers an explanation of how this is predicted to work (Figure 5). For example, PFC working memory cells do not fire during such tasks that do not require storage of visual information (Fuster, 1973; Kojima and Goldman-Rakic, 1984). Moreover, given the presentation of identical stimuli, neural selectivity in PFC depends on subsequent task demands (Warden and Miller, 2010). Imaging data show that success on working memory tasks covaries with an individual’s ability to selectively identify and store task-related stimuli from a larger sequence of stimuli (Awh and Vogel, 2008; McNab and Klingberg, 2008). Subliminal distracters can damage performance in attention tasks, but making distracters supra-threshold can improve performance deficits by facilitating the ability to filter them out (Tsushima et al., 2008). During a memory saccade task in which a salient distractor is flashed at a variable time and position during the memory delay, responses to the salient distractor are more strongly suppressed and correlated with performance in DLPFC than in LIP (Suzuki and Gottlieb, 2013).

In addition to this kind of task-sensitive filtering of individual items *before* they reach the working memory, a mechanistically distinct processes enables all the items that get through the filter to be stably stored *after* they reach the working memory; namely, keeping an SNr gate open to enable the recurrent excitatory connections within PFC to maintain working memory storage. Closing this SNr gate can rapidly reset, or delete, the entire stored sequence from working memory when there is an attention shift to do a different task.

### 7.3. Interacting Feature-Based Attention, Saccadic Choice, and Selective Working Memory Storage

The property of selective working memory storage clarifies the functional role of neurophysiological data about the role of VPA as “a source for feature-based attention” (Bichot et al., 2015, p. 832), notably why VPA cells selectively match desired combinations of object features, resonate with a target that matches these features, and activate an FEF positional representation that commands a saccade to the target. These properties were discovered when fixating monkeys were presented with a central cue object that defined a search target, followed by a delay during which the monkeys held a representation of the target in memory. Then an array of eight stimuli appeared which included the search target and seven distractors. The monkeys were rewarded for foveating and maintaining fixation on the target for 800 ms. While the monkeys performed, Bichot et al. (2015) simultaneously recorded from IT, VPA, and FEF in two monkeys, and VPS, VPA, and FEF in two other monkeys.

pART proposes the following mechanistic and functional explanation of how these cells interact together to enable matched objects to be selectively processed and stored by PFC (Figure 5): Both ITp (TEO) and ITa (TE) topographically project to PFC (Barbas and Pandya, 1989; Webster et al., 1994; Tanaka, 1996). The ITp projection is to VPA, whose cells, just like the ones in ITp (Tanaka, 1996), exhibit significant sensitivity to extrafoveal positions (Bichot et al., 2015). The ITa projections are to PRC and VPS, which in turn projects to VLPFC. In the data of Bichot et al. (2015), VPS had the largest spatial tuning curves of any cells in their data, consistent with ITa invariance properties.

Active VLPFC top-down signals project to both VPS and VPA, and learn modulatory top-down expectations when VPS and VPA cells are also active. In pART, these expectations obey the ART Matching Rule that is realized by a top-down, modulatory on-center, off-surround network.

VPA cells that receive a previously learned VLPFC-to-VPA prime are enhanced when an extrafoveal object matches its target features, and are suppressed when the object mismatches them, properties that are consistent with the ART Matching Rule. This enhanced VPA activity is sufficient to trigger an output signal to FEF at the corresponding FEF positional representation in FEF. This property is supported by Bichot et al. (2015) data showing VPA activating around 20 ms. before FEF does. FEF can then trigger a saccade to foveate the target. Because objects that mismatch the VPA expectation are inhibited, they are not foveated.

A similar match-mismatch dichotomy regulates the activity of VPS cells when they receive an active VLPFC-to-VPS prime. Their activity is enhanced when an ITa invariant object category matches their receptive field, and are suppressed by a mismatch, again consistent with the ART Matching Rule. When a match occurs, a synchronous VPS-ITa resonance develops that enables the category’s temporal order to be stored in VLPFC. This resonance can also propagate top-down through multiple cortical areas (e.g., ITa-ITp-V4-V2-V1 in Figure 5) and supports conscious recognition of the object.

## 8. Learning the Present Position in Space of a Navigator Using Grid Cells and Place Cells

**FIGURE 14:**
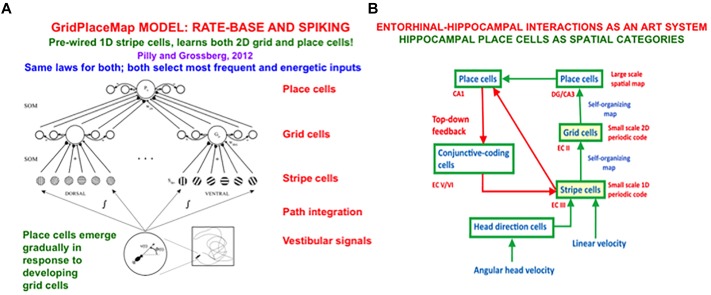
**(A)** The GridPlaceMap model circuit for spatial representation defines a hierarchy of self-organizing maps (SOM) that learns both grid cell and place cell maps. Stripe cells (*S_das_*) in the deeper layer of medial entorhinal cortex (MEC), self-organizing grid cells (*G_js_*) in layer II of MEC, and self-organizing place cells (*P_k_*) in hippocampal area CA3 learn to represent increasingly large spaces in response to internally generated signals based on vestibular linear velocity and angular velocity signals that are activated by navigational movements through the environment. Notice bigger stripe fields and spacings going from dorsal to ventral locations in the model organization. [Adapted with permission from Pilly and Grossberg (2012)]. **(B)** The hierarchy of self-organizing maps that learn grid cell and place cell maps is part of an ART system in which top-down feedback from place cells to stripe cells can dynamically stabilize the learned grid and place fields. See the text for details.

Section 5.2 noted that a representation of an animal’s Present Position Vector, or NET, as it navigates in space is derived in SOVEREIGN from an algorithm that computes a head/body turn angle as well the length of the next straight distance that is navigated. That section also noted that, in order for NET to be computed without algorithmic short cuts, an animal or animat needs to learn a representation of its present position in space as it navigates in different environments. The GridPlaceMap model of spatial navigation (Figure 14A) proposes how entorhinal grid cells and hippocampal place cells accomplish this as they are learned in a hierarchy of self-organizing maps. This model forms part of a larger entorhinal-hippocampal system that shows how learning of these maps may be dynamically stabilized by an entorhinal-hippocampal resonance (see section 6.11; Figure 14B; Grossberg and Pilly, 2014). This larger system explains why hippocampal place cells may be viewed as learned spatial categories in an entorhinal-hippocampal ART system that enables a stable computation of NET to be autonomously learned in a wide variety of navigated environments.

The GridPlaceMap model and its variants have explained and simulated many behavioral and neurobiological data about spatial navigation and how its circuits learn and remember (e.g., Grossberg and Pilly, 2012, 2014; Mhatre et al., 2012; Pilly and Grossberg, 2012; Grossberg, 2013; Grossberg et al., 2014). A comprehensive review of such data goes beyond the explanatory goals of the current exposition. Some basic facts are nonetheless worth mentioning here:

The model responds to realistic rat navigational trajectories by learning both grid cells with hexagonal grid firing fields of multiple spatial scales, and place cells with one or more firing fields, that match neurophysiological data about their development in juvenile rats. The fact that individual grid cells can fire at positions on a hexagonal lattice when rats navigate in an open field is one of the most remarkable facts in contemporary neuroscience (Hafting et al., 2005). The GridPlaceMap model and its variants show that this property emerges in a grid cell self-organizing map model (Figure 14) as a result of basic trigonometric properties of navigation in a two-dimensional space. The fact that hippocampal place cells may be viewed as learned spatial categories in an entorhinal-hippocampal ART system that are dynamically stabilized by top-down attention from hippocampal cortex to entorhinal cortex is supported by neurophysiological data from several labs (Morris and Frey, 1997; Kentros et al., 1998, 2004; Bonnevie et al., 2013).

Other properties of the GridPlaceMap model are also worth summarizing both because they are so parsimonious and data-predictive, and because they will simplify their embodiment in SOVEREIGN 2. For example, the *same* self-organizing map model equations can learn both grid cells and place cells. The different response properties seem to arise entirely due to their different stages of processing in a hierarchy of self-organizing maps (Figure 14B). In this hierarchy, hexagonal grid cell response fields are learned in response to stripe cells, which are derived from vestibular angular head velocity and linear velocity signals as realistic spatial trajectories are navigated (Figure 14). Place cells with unimodal response fields are learned in response to inputs from the emerging grid cells. Despite their very different response properties, both grid cells and place cells can develop by detecting, learning, and remembering the most frequent and energetic co-occurrences of their inputs. Because each place cell learns to respond to grid cells of several different spatial scales, the spatial scale of the resulting place cell is the *least common multiple* of the grid cell scales that input to it. Thus grid cells that respond on a centimeter scale can support learning of place cells that can represent spaces that are many meters in size.

Figure 14B also includes the known direct pathway from entorhinal cortex (EC111) to the hippocampal CA1 region that bypasses the grid cells. This pathway may learn place cells in CA1 with small spatial scales while, for example, rat pups are still in their nests. An explosion of coordinated grid cell and place cell development occurs as rats emerge from their nests (Langston et al., 2010; Wills et al., 2010), and presumably helps to learn the much larger spatial scales that are needed for adult spatial navigation.

Parsimonious properties also occur at the earliest stages of the GridPlaceMap model. For example, similar *ring attractor* networks are used to convert vestibular *angular velocity* signals into responses of head direction cells, and *linear velocity* signals into responses of stripe cells (Figure 14B). Both spatial and temporal learning in the entorhinal-hippocampal system seem to use homologous mechanisms to create a gradient from small to large scales along a dorsoventral axis. The temporal learning is the adaptively timed hippocampal learning that was described in sections 6.7 and 6.8. In particular, during both spatial and temporal learning, cells in different positions along the gradient respond at slower rates from dorsal to ventral. Spatial learning of grid cells and place cells along the dorsoventral axis passes through the *medial* entorhinal cortex to HIPPO, with the largest grid and place cell spatial scales occurring at ventral positions. Spatial learning hereby converts slower cell response rates into larger learned spatial scales. Temporal learning along the dorsoventral axis passes through the *lateral* entorhinal cortex to HIPPO, with the longest time intervals spanned at the most ventral positions in this gradient. Temporal learning uses spectrally timed conditioning with cells in the spectrum responding more slowly at more ventral positions (Figure 11C).

This computational homology provides a harmonious explanation of why both spatial and temporal representations occur in the entorhinal-hippocampal system. Many challenging neurophysiological data are explained by this homology between spatial learning in the medial entorhinal-hippocampal system and adaptively timed temporal learning in the lateral entorhinal-hippocampal system (e.g., Hargreaves et al., 2005; Aminoff et al., 2007; Kerr et al., 2007; Eichenbaum and Lipton, 2008; van Strien et al., 2009; Keene et al., 2016). When comparing these spatial and temporal circuits, the GridPlaceMap model is called *spectral spacing* to match the term spectral timing. The computational homology between them is called *neural relativity*.

The top-down hippocampus-to-entorhinal attentional network that stabilizes map learning uses the same ART Matching Rule that stabilizes learning of all ART circuits, including object categories learned via a feature-category resonance (Figure 7, 9). In the entorhinal-hippocampal system, this attentive matching process helps to explain neurobiological data about theta, beta, and gamma oscillations, such as, as mentioned above, why there is an Inverted-U through time in the power of beta oscillations when an animal first navigates a new maze (Berke et al., 2008; Grossberg, 2009a). Also explained are data about how hippocampal, septal, or acetylcholine inactivation may disrupt grid cell learning and performance.

## 9. Concluding Remarks

This article summarizes basic design principles, networks, and functional capabilities of the SOVEREIGN architecture (Gnadt and Grossberg, 2008) and outlines a major research program whereby additional brain mechanisms and psychological functions can be consistently added to create a SOVEREIGN2 architecture with much greater capabilities for autonomous adaptive navigation and goal-oriented cognition, emotion, and action in changing environments.

SOVEREIGN was designed to serve as an autonomous neural system for incrementally learning planned action sequences to navigate toward a rewarded goal. SOVEREIGN also illustrates how brains may, at several different organizational levels, regulate the balance between reactive and planned behaviors, and proposes how homologous circuit designs regulate spatial navigation and reaching behaviors. These capabilities were demonstrated by learning efficient routes whereby to navigate to a valued goal in a virtual reality environment.

Some of the designs in SOVEREIGN were realized algorithmically, and can be realized dynamically in SOVEREIGN2. Other processes that are needed to achieve a more comprehensive autonomous adaptive intelligence in an embodied mobile system were not included at all. This article summarizes neural models of important missing capabilities with enough detail to define a research program that that can consistently incorporate them into SOVEREIGN2. Missing designs occur across both the What and Where processing streams of SOVEREIGN (e.g., Figure 4).

In order to include these missing designs, SOVEREIGN2 embodies foundational brain design principles such as complementary computing, hierarchical resolution of uncertainty, and adaptive resonance that enable biological brains to realize their autonomous adaptive intelligence. Some of the missing designs in the What stream occur at early processing stages, such as visual boundary completion and surface filling-in. These processes require hierarchical resolution of uncertainty to be completed. How this occurs sheds light on deep computational reasons for how and why animals like humans and other primates become conscious in order to generate effective actions.

Other missing What stream processes occur at higher processing stages, such as autonomous learning of view-, position-, and size-invariant recognition categories. Such invariant learning requires modulatory interactions from parietal regions of the Where cortical stream to inferotemporal regions of the What cortical stream in order to ensure that only views of a single object get bound together by associative learning in a single invariant object category. The surface-shroud resonances that support invariant category learning also play a role in enabling social cognitive skills such as joint attention and imitation learning to occur between a teacher and a student who experience the world through different spatial perspectives.

Still higher levels of processing have parallel object and spatial processing systems in both the What and Where cortical streams. For example, prefrontal object and spatial working memories need to be able to selectively filter targets from distractors before storing them and their target positions in working memory. The filtering machinery that does this also allows attention to be paid to salient targets, and to use those targets to drive orienting movements toward them.

Cognitive-emotional circuits are needed to enhance predictions and actions that lead to valued outcomes, and to attenuate those that do not. In order to do this effectively, cognitive-emotional learning needs to be able to associate sensory and rewarding cues that are separated in time. Spectral timing circuits in the HIPPO help to support cognitive-emotional learning in inferotemporal-amygdala/hypothalamus-orbitofrontal circuits. These circuits, in turn, amplify or suppress cognitive and spatial working memory circuits and plans according to whether they generate successful goal-oriented actions or not.

Although looking and reaching behaviors can use target position and present position estimates that can both be directly computed from either external sensory cues or internally generated movement commands, navigational movements need more sophisticated networks to learn a navigator’s present position in space. Entorhinal grid cells and hippocampal place cells interact to incrementally learn place cells that can represent spatial scales that are sufficiently large to support navigation in ecologically relevant spaces. These learned spatial categories are dynamically stabilized using the same Adaptive Resonance Theory, or ART, Matching Rule that is found in the resonant dynamics of many of the missing competences from which SOVEREIGN2 can benefit.

These resonances include feature-category resonances, surface-shroud resonances, cognitive-emotional resonances, entorhinal-hippocampal resonances, and item-list resonances. All of these resonances help to dynamically stabilize the learned memories of their respective networks, and thereby enable them to successfully operate in open-ended non-stationary environments without experiencing the learning and forgetting problems, notably catastrophic forgetting, that plagues all algorithms of back propagation type, including the currently popular and useful Deep Learning algorithms, and Bayesian Explaining Away algorithms, among others.

## Author Contributions

The author confirms being the sole contributor of this work and has approved it for publication.

## Conflict of Interest Statement

The author declares that the research was conducted in the absence of any commercial or financial relationships that could be construed as a potential conflict of interest.

## Footnotes

1 https://www.darpa.mil/program/explainable-artificial-intelligence

## Supplementary Material

FIGURE S1 Smooth pursuit of a target moving with a fixed speed and direction creates retinal slip signals on the retina until the target is foveated, as well as background motion signals in the opposite direction. As the target is acquired, the background motion signals increase, and can maintain predictive pursuit that maintains the target on the fovea. See the text for details. [Reprinted with permission from Pack et al. (2001)].

FIGURE S2 **(a)** A leftward eye movement channel. All connections are excitatory. The retinal image is processed by two types of cells in MT. MT cells with inhibitory surrounds (MT^-^) connect to MSTv cells, with MT cells preferring greater speeds weighted more heavily. MT cells with excitatory surrounds (MT^+^) connect to MSTd cells. MSTv cells have excitatory connections with MSTd cells that prefer opposite directions. MSTv cells drive pursuit eye movements in their preferred direction, and the resulting eye velocity is fed back to MSTv and MSTd cells (thick arrows). Leftward eye rotation causes rightward retinal motion of the background. The MT and MST cells are drawn so as to approximate their relative receptive field sizes. **(b)** Model MST connectivity. Excitatory connections are shown by solid lines. Inhibitory connections are indicated by dashed lines. Thick line emanating from the pursuit pathway indicate efference copy inputs. The leftward eye movement channel consists of an MSTv cell preferring leftward motion and an MSTd cell preferring rightward motion, and receives an efference copy signaling leftward eye movement. The rightward eye channel is defined analogously. [Reprinted with permission from Pack et al. (2001)].

FIGURE S3 In this figure, black boxes denote areas belonging to the saccadic eye movement system (SAC), white boxes the smooth pursuit eye movement system (SPEM), and gray boxes, both systems. The abbreviations for the different brain regions are: LIP, lateral intra-parietal area; FPA, frontal pursuit area; MST, middle superior temporal area; MT, middle temporal area; FEF, frontal eye fields; NRTP, nucleus reticularis tegmenti pontis; DLPN, dorso-lateral pontine nuclei; SC, superior colliculus; CBM, cerebellum; MVN/rLVN, medial and rostro-lateral vestibular nuclei; PPRF, a peri-pontine reticular formation; TN, tonic neurons. Although an analysis of how this system works is beyond the scope of this article, the macrocircuit does serve as a reminder that seemingly effortless behavioral competences are often emergent properties of beautifully coordinated brain dynamics among multiple brain regions with different functional roles to play [Reprinted with permission from Grossberg et al. (2012)].

FIGURE S4 Two views of the eye and retina. The top image shows a drawing of a cross-sectional cut through the eye showing the retinal veins occluding the light coming into the pupil before it reaches the photoreceptors. The photoreceptors send axons to the brain via the optic nerve which, as seen in the bottom image of a top-down view of retina, creates a blind spot that is comparable in size to the fovea [Adapted with permission from Kolb, Fernandez, and Anderson (http://retina.umh.es/Webvision/sretina.html)].

FIGURE S5 This image emphasizes that, even the retinal image of a simple object like a line can be occluded in multiple places by retinal veins and the blind spot, thereby creating multiple positions along the line that do not provide reliable inputs to the brain for directing actions to those positions.
