# Supplementary figures and images for: The Embodied Brain of SOVEREIGN2: From Space-Variant Conscious Percepts During Visual Search and Navigation to Learning Invariant Object Categories and Cognitive-Emotional Plans for Acquiring Valued Goals
Source: Front Comput Neurosci. 2019 Jun 25;13:36. doi: 10.3389/fncom.2019.00036 (PMC6620614; doi:10.3389/fncom.2019.00036)

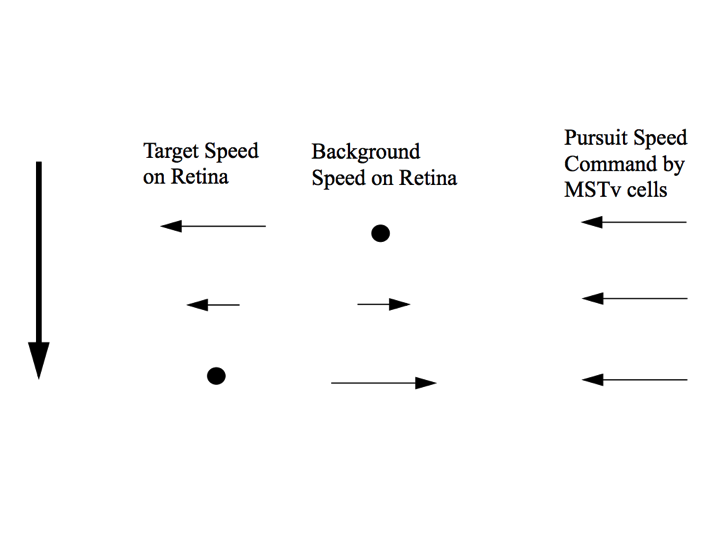

Supplement: FIGURE S1 — Smooth pursuit of a target moving with a fixed speed and direction creates retinal slip signals on the retina until the target is foveated, as well as background motion signals in the opposite direction. As the target is acquired, the background motion signals increase, and can maintain predictive pursuit that maintains the target on the fovea. See the text for details. [Reprinted with permission from Pack et al. (2001)]. [file Image_1.tiff]

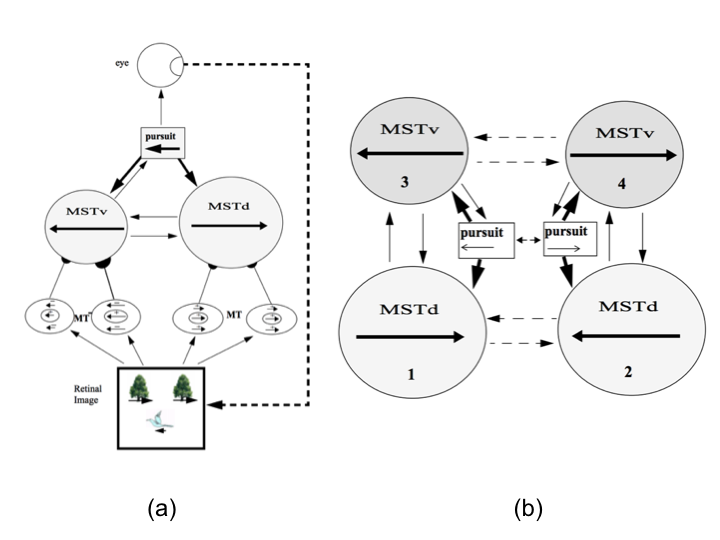

Supplement: FIGURE S2 — (a) A leftward eye movement channel. All connections are excitatory. The retinal image is processed by two types of cells in MT. MT cells with inhibitory surrounds (MT-) connect to MSTv cells, with MT cells preferring greater speeds weighted more heavily. MT cells with excitatory surrounds (MT+) connect to MSTd cells. MSTv cells have excitatory connections with MSTd cells that prefer opposite directions. MSTv cells drive pursuit eye movements in their preferred direction, and the resulting eye velocity is fed back to MSTv and MSTd cells (thick arrows). Leftward eye rotation causes rightward retinal motion of the background. The MT and MST cells are drawn so as to approximate their relative receptive field sizes. (b) Model MST connectivity. Excitatory connections are shown by solid lines. Inhibitory connections are indicated by dashed lines. Thick line emanating from the pursuit pathway indicate efference copy inputs. The leftward eye movement channel consists of an MSTv cell preferring leftward motion and an MSTd cell preferring rightward motion, and receives an efference copy signaling leftward eye movement. The rightward eye channel is defined analogously. [Reprinted with permission from Pack et al. (2001)]. [file Image_2.tiff]

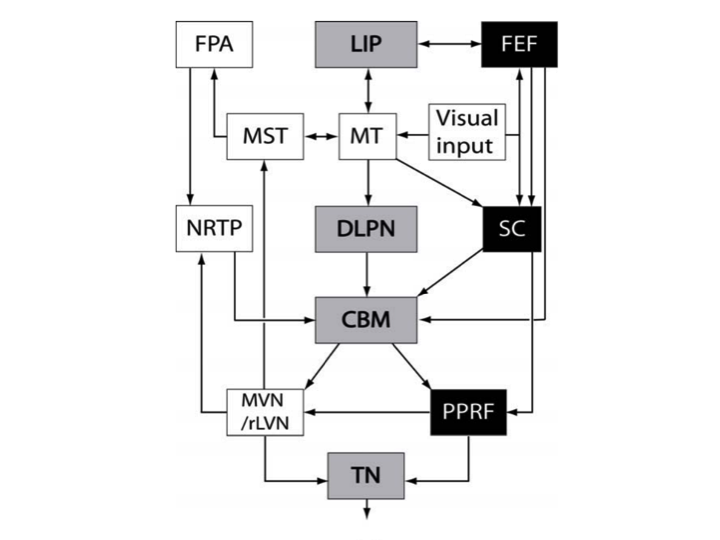

Supplement: FIGURE S3 — In this figure, black boxes denote areas belonging to the saccadic eye movement system (SAC), white boxes the smooth pursuit eye movement system (SPEM), and gray boxes, both systems. The abbreviations for the different brain regions are: LIP, lateral intra-parietal area; FPA, frontal pursuit area; MST, middle superior temporal area; MT, middle temporal area; FEF, frontal eye fields; NRTP, nucleus reticularis tegmenti pontis; DLPN, dorso-lateral pontine nuclei; SC, superior colliculus; CBM, cerebellum; MVN/rLVN, medial and rostro-lateral vestibular nuclei; PPRF, a peri-pontine reticular formation; TN, tonic neurons. Although an analysis of how this system works is beyond the scope of this article, the macrocircuit does serve as a reminder that seemingly effortless behavioral competences are often emergent properties of beautifully coordinated brain dynamics among multiple brain regions with different functional roles to play [Reprinted with permission from Grossberg et al. (2012)]. [file Image_3.tiff]

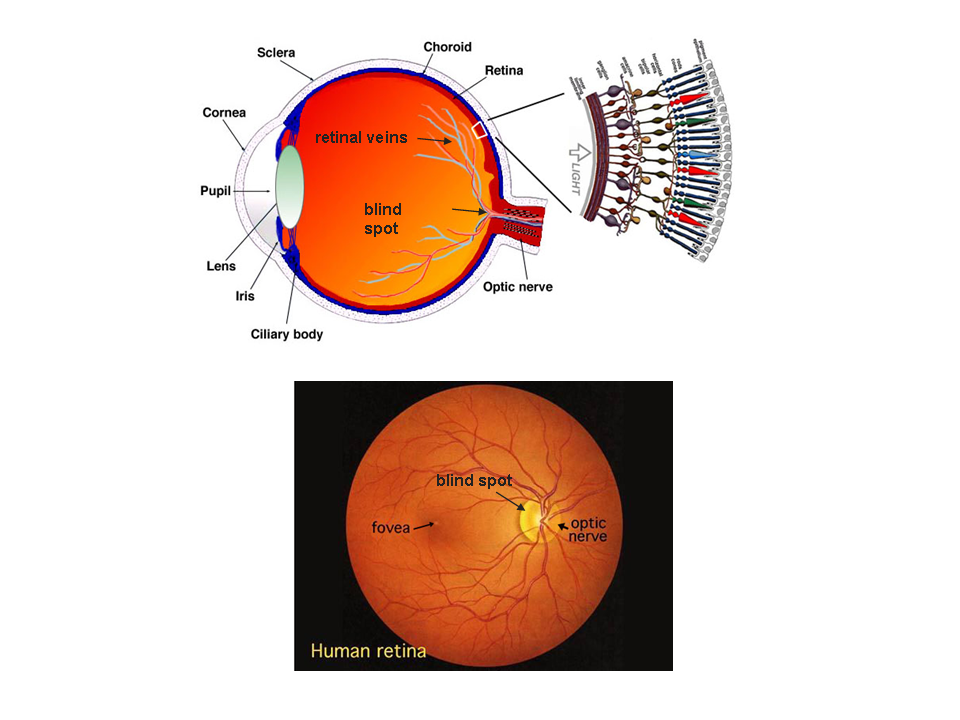

Supplement: FIGURE S4 — Two views of the eye and retina. The top image shows a drawing of a cross-sectional cut through the eye showing the retinal veins occluding the light coming into the pupil before it reaches the photoreceptors. The photoreceptors send axons to the brain via the optic nerve which, as seen in the bottom image of a top-down view of retina, creates a blind spot that is comparable in size to the fovea [Adapted with permission from Kolb, Fernandez, and Anderson (http://retina.umh.es/Webvision/sretina.html)]. [file Image_4.tif]

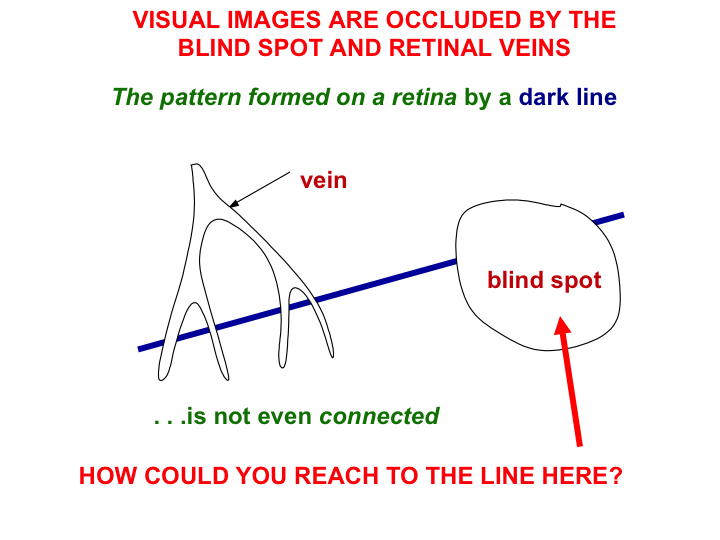

Supplement: FIGURE S5 — This image emphasizes that, even the retinal image of a simple object like a line can be occluded in multiple places by retinal veins and the blind spot, thereby creating multiple positions along the line that do not provide reliable inputs to the brain for directing actions to those positions. [file Image_5.tiff]
